# Retrotransposon‐based genetic diversity of *Deschampsia antarctica* Desv. from King George Island (Maritime Antarctic)

**DOI:** 10.1002/ece3.7095

**Published:** 2020-12-16

**Authors:** Piotr Androsiuk, Katarzyna J. Chwedorzewska, Justyna Dulska, Sylwia Milarska, Irena Giełwanowska

**Affiliations:** ^1^ Department of Plant Physiology, Genetics and Biotechnology Faculty of Biology and Biotechnology University of Warmia and Mazury in Olsztyn Olsztyn Poland; ^2^ Department of Agronomy Warsaw University of Life Sciences‐SGGW Warsaw Poland

**Keywords:** Antarctic hairgrass, Antarctica, genetic diversity, iPBS, King George Island

## Abstract

*Deschampsia antarctica* Desv. can be found in diverse Antarctic habitats which may vary considerably in terms of environmental conditions and soil properties. As a result, the species is characterized by wide ecotypic variation in terms of both morphological and anatomical traits. The species is a unique example of an organism that can successfully colonize inhospitable regions due to its phenomenal ability to adapt to both the local mosaic of microhabitats and to general climatic fluctuations. For this reason, *D. antarctica* has been widely investigated in studies analyzing morphophysiological and biochemical responses to various abiotic stresses (frost, drought, salinity, increased UV radiation). However, there is little evidence to indicate whether the observed polymorphism is accompanied by the corresponding genetic variation. In the present study, retrotransposon‐based iPBS markers were used to trace the genetic variation of *D. antarctica* collected in nine sites of the Arctowski oasis on King George Island (Western Antarctic). The genotyping of 165 individuals from nine populations with seven iPBS primers revealed 125 amplification products, 15 of which (12%) were polymorphic, with an average of 5.6% polymorphic fragments per population. Only one of the polymorphic fragments, observed in population 6, was represented as a private band. The analyzed specimens were characterized by low genetic diversity (uH_e_ = 0.021, *I* = 0.030) and high population differentiation (*F*_ST_ = 0.4874). An analysis of Fu's *F*_S_ statistics and mismatch distribution in most populations (excluding population 2, 6 and 9) revealed demographic/spatial expansion, whereas significant traces of reduction in effective population size were found in three populations (1, 3 and 5). The iPBS markers revealed genetic polymorphism of *D. antarctica*, which could be attributed to the mobilization of random transposable elements, unique features of reproductive biology, and/or geographic location of the examined populations.

## Graphical abstract

Deschampsia antarctica is among the two angiosperms considered as a native for the Antarctic. The retrotransposon‐based iPBS markers applied in the study revealed low genetic variation of the species accompanied by surprisingly high population differentiation.

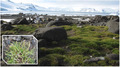

## 1 INTRODUCTION

**FIGURE 1:**
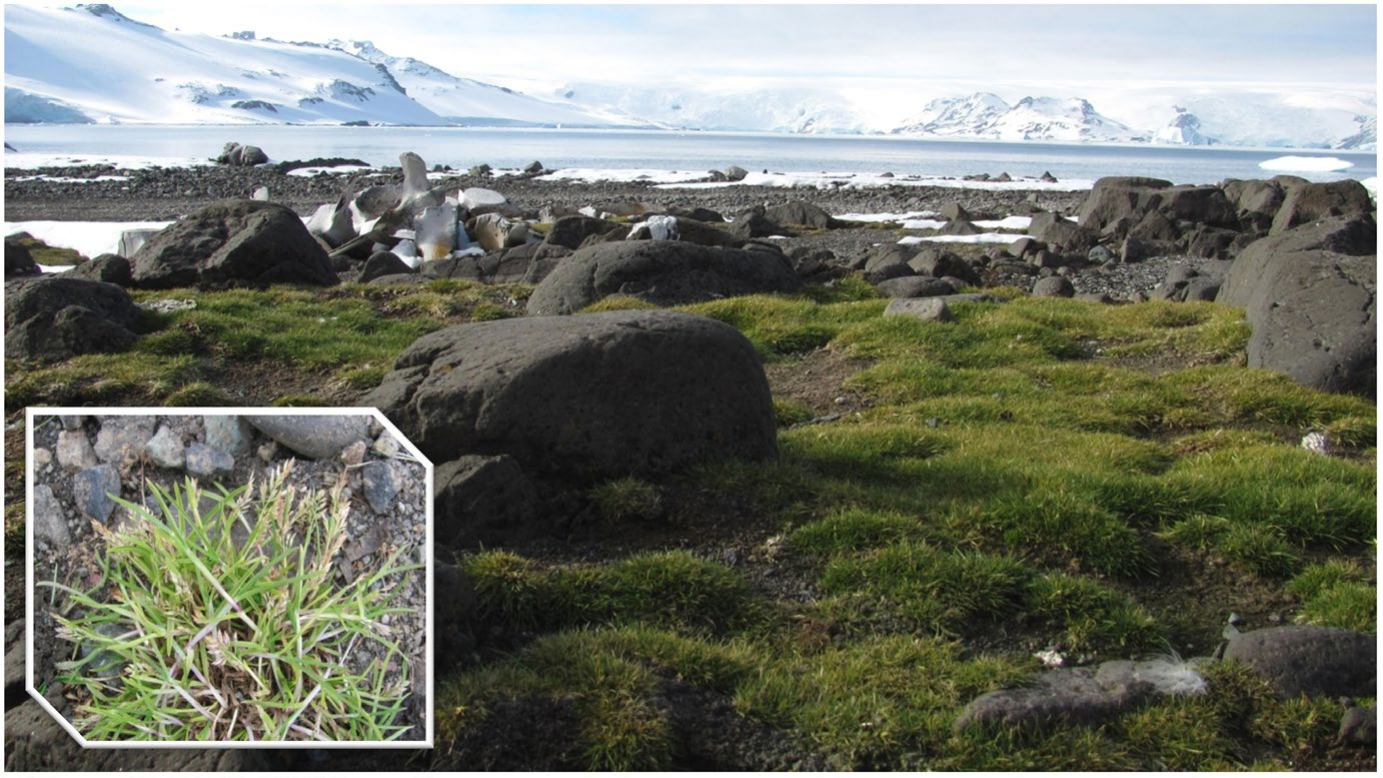
*Deschampsia antarctica* on the King George Island, South Shetland Islands. (fot. by I. Giełwanowska)

Antarctic terrestrial biota occurs mainly in small, isolated ice‐free areas in the coastal zones of the Maritime Antarctic (Lee et al., 2017). The development of terrestrial ecosystems is generally limited by environmental factors such as low temperature, intermittent water supply, highly seasonal light regime, ground‐level wind speed, uneven distribution of nutrients, high salinity in locations with strong marine influence, and seasonal environmental stresses (Beyer et al., 2000; Convey & Peck, 2019). Soil properties are an important abiotic factor in the Antarctic ecosystem (Lachacz et al., 2018; Sierakowski et al., 2017; Znój et al., 2017). The Antarctic terrestrial ecosystem is a mosaic of microhabitats that differ in the availability of nutrients, water conditions, the influence of salty aerosols, and differences in exposition. As a result, ice‐free areas became colonized by highly heterogeneous and discontinuous plant communities that are interspersed with bare ground and are dominated by cryptogams. The only two native angiosperms are *Deschampsia antarctica* Desv. (*Poaceae*) (Antarctic hairgrass) (Figure 1) and *Colobanthus quitensis* (Kunth) Bartl. (*Caryophyllaceae*) (Ochyra et al., 2008; Olech, 2004). The distribution of *D. antarctica* is restricted to the Maritime Antarctic, including the west coast of the Antarctic Peninsula, its offshore islands and the South Sandwich, South Orkney, and South Shetland Islands. The species is also commonly encountered on sub‐Antarctic islands such as South Georgia Island, Heard Island, Crozet Islands, and the Kerguelen Archipelago in the Indian Ocean. Outside the sub‐Antarctic region, *D. antarctica* is found on the Falkland Islands and in South America in Tierra del Fuego and the Andes up to a latitude of around 34 degrees south (Convey, 1996).

Genetic studies demonstrated very low levels of genetic variability in *D. antarctica,* even in populations separated by a considerable distance (Holderegger et al., 2003; Chwedorzewska & Bednarek, 2008, 2011; van de Wouw et al., 2008). Despite the above, *D. antarctica* is characterized by remarkable ecotypic variation and colonizes a wide range of habitats, from mineral soils to organic soils and eroded pits, from extremely dry windswept fellfields to waterlogged seepage areas that are occasionally inundated by sea water (Lewis‐Smith, 2003), as well as nutrient‐deficient sites or habitats that are enriched mainly with ammonium and nitrates by animals and sea spray (Lewis‐Smith, 2003; Nędzarek & Chwedorzewska, 2004). Even a small shift in environmental conditions such as exposition or distance from the source of nutrients (bird colonies) and sea can lead to the development of different terrestrial communities. *Deschampsia antarctica* populations originating from such sites differ significantly in morphological and anatomical traits (Chwedorzewska et al., 2008; Corner, 1971; Giełwanowska, 2003a,b).

Resistance to severe and diverse physiological stresses is essential for survival in the harsh Antarctic climate (Clemente‐Moreno et al., 2020). Stressors can induce a flexible response from an organism and lead to the development of a new phenotype. Environmentally induced genetic changes in transposable elements (TEs) are one of such mechanisms (Kalendar et al., 2000; Piacentini et al., 2014). Transposable elements are capable of changing their location and/or copy numbers, and they play an important role in the evolution of the plant genome (Finnegan, 1989). Transcriptional activation of TEs has been observed in many plant species that are exposed to various abiotic and biotic stressors (Moreau‐Mhiri et al., 1996; Takeda et al., 1998; Voronova et al., 2011), and it is regarded as a key mechanism that is responsible for genome plasticity under changing environmental conditions (Schrader et al., 2014). Intense stress may facilitate rapid changes in the structure, organization, and function of the genome through interactions with TEs, especially in populations with low genetic diversity (Stapley et al., 2015). The evolution of environmentally induced advantageous phenotypes through epigenetic mechanisms could be an immediate adaptive process, followed by TE‐induced genotypic changes that make these phenotypic variants heritable through the germline. Transposable elements could play different roles on the timescale of ecological variation (Pimpinelli & Piacentini, 2020), such as those related to diverse stress conditions in Antarctic habitats. The activation of TEs can induce genetic variability in response to environmental changes. Transposable elements also exert selective pressure on another genetic elements, thus contributing to rapid evolutionary processes and adaptation to local conditions.

Transposable elements are highly abundant and diverse mobile genetic elements that constitute up to 90% of eukaryotic genomes (San Miguel et al., 1996). Many features of TEs, such as their ubiquity, abundance, and dispersion in the eukaryotic genome, make them an attractive target for molecular marker systems (Kalendar et al., 2019). Various approaches have been proposed to explore polymorphisms in TE insertion patterns, including conventional or anchored PCR, and quantitative or digital PCR with primers designed for the 5' or 3' junction (Kalendar et al., 2019). The main drawback of TE‐based molecular markers techniques is the need for sequence information in designing element‐specific primers. In species where genomic data are scarce or absent, genetic polymorphism resulting from TE mobility can be detected based on their conserved sequences. The Inter Primer Binding Sequence (iPBS) technique developed by Kalendar et al. (2010) relies on the highly conserved domain of LTR retrotransposons for primer binding. This method proved to be a valuable tool for assessing retrotransposon‐based genetic variation in guava (Mehmood et al., 2016), beech, chestnut, and oak (Coutinho et al., 2018), barley (Bonchev et al., 2019) and *Colobanthus quitensis* populations from a wide geographic range (Koc et al., 2018), or sites exposed to diverse abiotic conditions such as the Maritime Antarctic (Androsiuk et al., 2015).

In the present study, iPBS markers were used to assess DNA polymorphism and genetic relationships between *D. antarctica* populations from patchy habitats of the Arctowski oasis (King George Island, South Shetlands, Antarctic).

## 2 MATERIALS AND METHODS

### 2.1 Material

**FIGURE 2:**
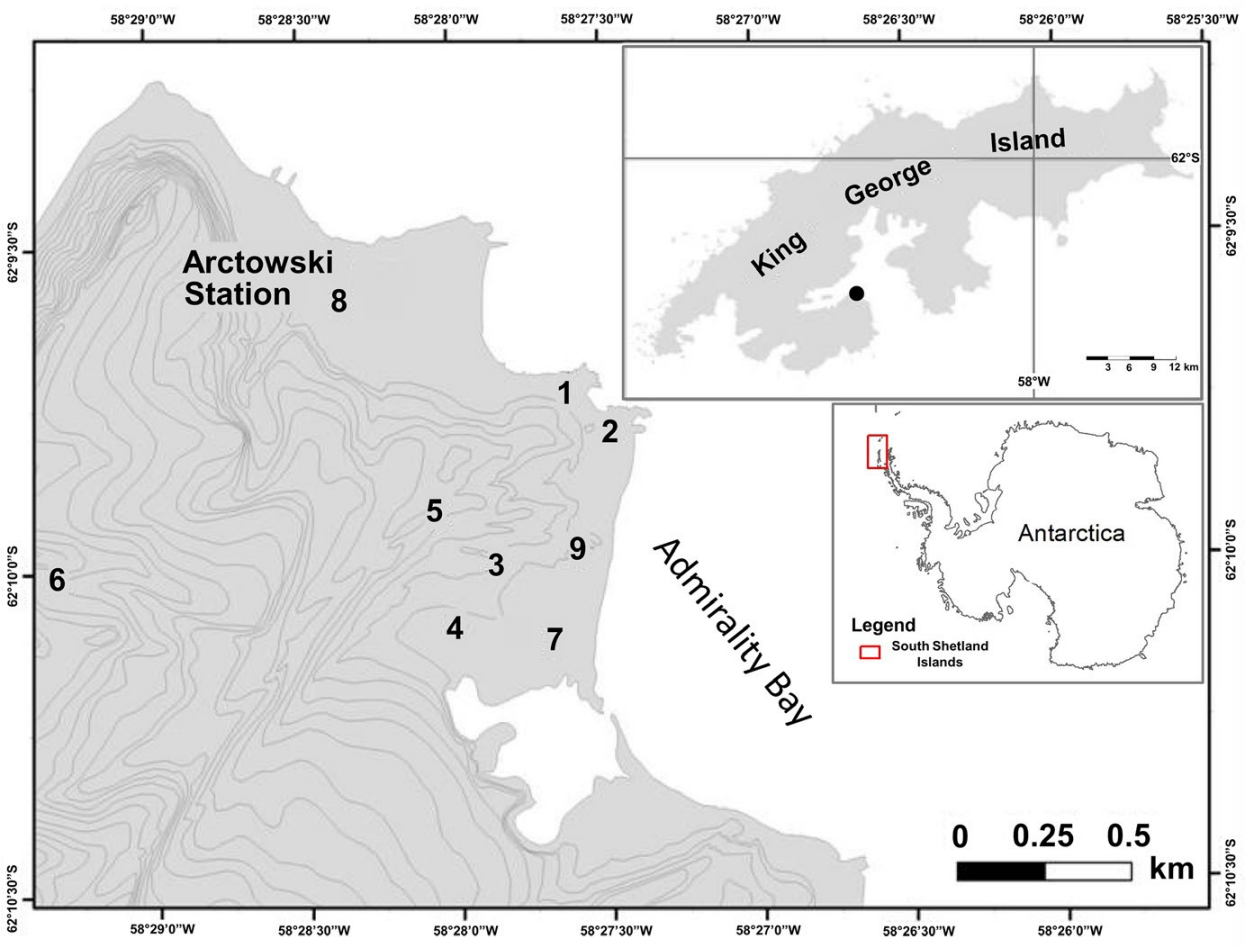
Study area showing sampling sites of *Deschampsia antarctica* on King George Island and contour map of Antarctica with marked location of South Shetland Islands. Numbers of populations according to Table 1

A total of 165 *D. antarctica* plants sampled in nine sites with different microclimatic conditions and soil properties in the Arctowski oasis were subjected to molecular analyses (Figure 2, Table 1, Table S1). *D. antarctica* fresh, healthy leaves were stored at −20°C immediately after sampling in 2010. The population from each sampling site was represented by 10 to 33 specimens. The samples were not equal in size because the number of individuals was very small in some sites. In addition, many dry and exposed sites featured small individuals with high necromass content; therefore, sufficient quantities of fresh material for DNA extraction were difficult to collect.

**TABLE 1:** The origin of *Deschampsia antarctica* populations used in the study

No.	Vegetation	Edaphic characteristics	Distance from the sea [m]	Altitude [m a.s.l.]	Location	Number of individuals
1.	The area has abundant plant cover, mainly *D. antarctica, C. quitensis,* and *Prasiola crispa,* a green algae that develops rapidly during the growing season; ornithocoprophilous lichen species grow on rocks	Basalt rock outcrops where soils developed only in rock crevices; soil has high organic matter content originating mainly from fresh penguin guano; the habitat is humid and sheltered from the wind; area trampled by birds.	100–120	10	62.1621S, 58.4606W	14
2.	Many lichen species, including halophilous species *Verrucaria tesselatula. D. antarctica* and *C. quitensis* (less frequent than in site 1), as well as *Prasiola crispa*	Within the range of ocean waves during stormy weather; soils were classified as Eutric Skeletic Nudilithic Leptosols (Arenic, Humic, Ornithic, Protic); soil has high organic matter content originating mainly from fresh penguin guano; humid and exposed habitat	1–2	0.5–1.0	62.1629S, 58.4567W	21
3.	The site is covered by the Antarctic tundra; mosses, lichens and two species of flowering plants, *D. antarctica* and *C. quitensis,* were identified; plants are short (2.5–6.5 cm)	The oldest moraine of the Ecology Glacier; sloping edge of a relatively old fluted moraine; strongly graveled soils; habitat dry and exposed; the site occupies a former penguin rookery, with relict bird influences; within the range of sea water aerosols during stormy weather; soil was classified as Skeletic Protic Turbic Cryosol (Arenic, Eutric, Humic, Ornithic).	400	40	62.1639S, 58.4599W	21
4.	Lichens, *D. antarctica* and *C. quitensis* were identified, but plant cover was less extensive; flowering plants have a height of 3.5–7.5 cm; plant growth is relatively rapid	A moraine of the Ecology Glacier; dry and exposed habitat with a minor influence of penguin and seal rookeries; site 4 is similar to site 3, but younger; soil was classified as Eutric Protic Skeletic Regosol (Loamic, Turbic).	400	35	62.1645S, 58.4603W	11
5.	Typical Antarctic tundra; mosses, lichens and flowering plants form a dense carpet; flowering plants grow slowly and reach 1.5–2.5 cm in height	Near the grave of W. Puchalski; gravelly material is water permeable and well drained; old penguin rookery with relict ornithogenic soil; habitat dry and exposed; soil was classified as Skeletic Protic Turbic Cryosol (Arenic, Dystric, Humic, Ornithic).	500	110	62.1635S, 58.4626W	33
6.	The proportion of flowering plants is small, with a predominance of lichens	Near the Jersak Hills glacier; far from the coast (700 m); basalt rock outcrops with scree debris below; soil was classified as Eutric Protic Skeletic Leptic Regosol (Arenic, Humic, Turbic); habitat dry and exposed; soil has low nutrient and organic matter content	700	200	62.1649S, 58.4874W	10
7.	Numerous rocks with the smallest plant cover in all studied sites; plant age was determined based on flowering tussocks: *Colobanthus quitensis* plants appeared in this location approx. 10 years ago, and *Deschampsia antarctica* plants appeared in this location approx. 3–5 years ago	The youngest fluted moraine of the Ecology Glacier with rich petrographic composition; close to a fresh water lagoon from a melting glacier; very high moisture content due to the direct influence of sea water; minor influence of penguin and seal rookeries; soil was classified as Eutric Protic Skeletic Protic Regosol (Loamic).	20	0.5	62.1682S, 58.4622W	16
8.	Two native flowering plants, an invasive grass species (*Poa annua*) and chlorophyte algae (*Prasiola crispa*)	The area of Henryk Arctowski Station; the ground was transformed due to continuous human influence (road, water tank); humid habitat sheltered from the wind; sea sands and fluvioglacial sands were mechanically altered; vegetation cover is limited due to mechanical impact exerted by people and vehicles; soil was classified as Skeletic Eutric Fluvisol (Arenic).	50	0.5	62.1598S, 58.4759W	27
9.	Mostly lichens, mosses and scattered specimens of *D. antarctica* and *C. quitensis*, excluding the area surrounding Arctowski Station (site 8)	Mouth of the Ornithologists Creek; fertile and moist soil supplied with both fresh water from the Ornithologists Creek and sea water which is accumulated for several hours after a storm; influence of the penguin colony (guano is the main source of organic matter); human impact on soil and vegetation is minimal, limited to occasional trampling on routes to study sites	30–40	1.0	62.1658S, 58.4589W	12

### 2.2 Molecular analyses

DNA was extracted from individuals representing each population with the Syngen Plant DNA Mini Kit. The quality of DNA was verified on 1% agarose, and the purity of DNA samples was assessed spectrophotometrically.

The entire *D. antarctica* collection was genotyped with iPBS primers. According to the procedure described by Kalendar et al. (2010), 20 iPBS primers were initially screened. Seven iPBS primers which produced clearly identifiable and repeatable polymorphic bands were selected for further analyses (Table 2). The reproducibility of primer band profiles was verified based on comparison of the electrophoretic profiles of randomly selected *D. antarctica* samples. Data were generated and compared in two replicates. Gels were then checked to identify iPBS amplicons (bands) in one or both replicates. Seven iPBS primers were used individually in a polymerase chain reaction (PCR) according to the protocol described by Kalendar et al. (2010) with some modifications (Androsiuk et al., 2015; Koc et al., 2018). The reaction conditions for each primer (temperature of primer hybridization) were determined empirically. The amplification products were analyzed by electrophoresis on 1.5% (w/v) agarose gels with 1x TBE electrophoresis buffer at 100 V for 2 hr and were visualized by staining with 0.5 μg/ml ethidium bromide.

**TABLE 2:** iPBS primers applied in the study and their specification

Primer	Sequence (5’→3’)	Annealing temp. (°C)	No of scored bands	No of polymorphic bands
2074	GCTCTGATACCA	52	18	1
2085	ATGCCGATACCA	51	17	3
2,224	ATCCTGGCAATGGAACCA`	52	16	2
2,251	GAACAGGCGATGATACCA3`	55	22	2
2,253	TCGAGGCTCTAGATACCA3`	53	24	2
2,374	CCCAGCAAACCA	55	14	1
2,376	TAGATGGCACCA	51	14	4
Total			125	15

### 2.3 iPBS data processing

All bands that were reliably identified across the studied individuals were scored as either present (1) or absent (0) across genotypes and treated as single dominant loci. Based on the obtained binary matrix of amplification products (Table S2), the following genetic parameters were estimated with the use of GenAlEx 6.5 software (Peakall & Smouse, 2006, 2012): total number of bands per population (*N_B_*), percentage of polymorphic bands (*P*), Shannon's Information Index (*I*), unbiased expected heterozygosity (uH_e_), and Nei's genetic distance (*D_N_*) (Nei, 1972). The genetic subdivision patterns of the analyzed *D. antarctica* populations were investigated by principal coordinate analysis (PCoA) based on *D_N_* values in GenAlEx 6.5. The genetic structure of the studied populations was inferred based on Bayesian model‐based clustering method implemented in STRUCTURE ver. 2.3.4. (Pritchard et al., 2000). The model assigns individual multilocus genotypes probabilistically to a user‐defined number of clusters (*K*), achieving linkage equilibrium within clusters. We performed 10 replicate runs for each *K*, ranging from 1 to 9, 500, 000 Markov Chain Monte Carlo repetitions and a burn‐in period of 500, 000. During the analysis, admixture model was used without any prior information on the original populations. To determine the optimal number of clusters, an ad hoc statistic ΔK was used (Evanno et al., 2005), estimated in Structure Harvester ver.0.6.94 (Earl & Vonholdt, 2012). Analysis of molecular variance (AMOVA) was also performed. In the analysis, the iPBS data were treated as haplotypic and composed of a combination of alleles at one or several loci (Excoffier et al., 2005). The significance of fixation indices was tested using a nonparametric permutation approach (Excoffier et al., 1992). The estimation of *F*_ST_ and AMOVA were performed using Arlequin 3.5 software (Excoffier et al., 2005). Additionally, with the use of Hickory v.1.1 package (Holsinger & Lewis, 2003), two alternative *F*_ST_ estimates were calculated: Gst‐B (Bayesian analog of Nei's *G*_ST_; Holsinger, 1999) and *θ* ^(^ *^III^* ^)^ which corresponds to a scaled allele frequency variance, where the variance is measured among contemporaneous populations (Song et al., 2006).

The possible effects of increased spatial distance and environmental heterogeneity on gene flow and genetic structure of the studied *D. antarctica* populations were also estimated. Spatial genetic structure was investigated by testing the significance of isolation by distance (IBD) in the Mantel test with 9,999 permutations of the relationship between the matrix of pairwise *F*_ST_/(1−*F*_ST_) and the matrix of log‐transformed geographic distances between populations (Rousset, 1997). The Mantel test with 9,999 permutations was also performed to compare the matrix of log‐transformed geographic distances between populations and the matrix of environmental distances to determine the degree of isolation by environment (IBE) of *D. antarctica* populations. Pairwise environmental distances (Euclidean distances) were calculated between the studied sampling sites based on the previously described data concerning general soil properties and nutrient content (Koc et al., 2018). The matrix containing pairwise environmental distances was standardized before the Mantel test based on the approach described by Nanninga et al. (2014). The pairwise environmental distance matrix was calculated and standardized in Statistica 12 software (StatSoft, Inc.). Sampling site 9 was not analyzed in the cited paper; therefore, only sites 1–8 were included in the IBE analysis. The Mantel test was performed in GenAlEx 6.5.

Tajima's *D*, Fu's *F*_S_ neutrality test, mismatch distribution, and the demographic processes affecting populations were estimated in Arlequin 3.5. Recent population history was inferred by examining the departure from the drift–mutation equilibrium based on allele frequencies in the BOTTLENECK v. 1.2.02 program (Cornuet & Luikart, 1996; Piry et al., 1999) for each population. In populations that have experienced a recent reduction in effective size, the value of *H*_e_ exceeds the heterozygosity expected at mutation–drift equilibrium. This effect was studied with the use of dominant markers in the infinite allele model (IAM) to test the mutation–drift hypothesis against the bottleneck hypothesis (Tero et al., 2003). The significance of potential bottleneck was estimated in the Sign test, the Standardized Differences test, and the one‐tailed Wilcoxon sign‐rank test for heterozygosity excess.

## 3 RESULTS

### 3.1 Efficiency of iPBS primers

The genotyping of *D. antarctica* populations with 7 iPBS primers supported the identification of 125 amplification products (bands). The highest number of 24 bands was revealed by primer 2,253, and the lowest number of amplification products (14) was obtained for primers 2,374 and 2,376. An average of 17.86 bands was obtained per primer. Fifteen of the identified loci (12%) were polymorphic (Table 2). A detailed analysis of genotypic data revealed one amplification product which could be represented as a potential private band for population 6. The band was amplified with primer 2,253, but it was identified in only one of 10 individuals.

### 3.2 Genetic diversity and differentiation

The iPBS markers revealed the presence of genetic polymorphism between individuals within a population as well as genetic variation between populations (Table 3). The number of iPBS amplification products (bands) ranged from 120 in population 2 to 124 in populations 5 and 6. The polymorphic rate was highest in population 5 (7%) and lowest in populations 2, 6, and 9 (5%). Genetic variation was assessed based on the values of Shannon's Information Index and unbiased expected heterozygosity, and both values were highest in population 5 and lowest in population 9.

**TABLE 3:** Population genetic characteristics for analyzed populations of *Deschampsia antarctica*

Populations	*N_B_*	*P*	*I*	uH_e_ ± *SE*
1	122	6.40	0.041	0.030 ± 0.010
2	120	4.80	0.023	0.016 ± 0.010
3	122	5.60	0.032	0.022 ± 0.009
4	123	5.60	0.028	0.020 ± 0.008
5	124	7.20	0.044	0.031 ± 0.010
6	124	4.80	0.024	0.017 ± 0.007
7	123	5.60	0.026	0.017 ± 0.007
8	123	5.60	0.029	0.020 ± 0.008
9	122	4.80	0.021	0.014 ± 0.007
Average over loci and populations	122.55	5.60	0.030	0.021

Abbreviations: *N_B_*, number of bands; *P*, percentage of polymorphic bands; *I*, Shannon's Information Index; uH_e_, unbiased expected heterozygosity with standard error (*SE*).

The results of AMOVA revealed that 51% of the identified genetic variation occurred between individuals within populations, whereas the remaining 49% was attributed to variation among populations (Table 4). High genetic variation between population was consistent with the *F*_ST_ value (0.4874), as well as Gst‐B (0.4349). The *θ* ^(^ *^III^* ^)^ estimate, though lower in value, has reasonably similar magnitude (0.3703).

**TABLE 4:** Partitioning of diversity found in *Deschampsia antarctica* from all analyzed populations using AMOVA (*F*_ST_ = 0.4874)

Source of variation	Degrees of freedom	Sum of squares	Variance components	Percentage of variation
Among populations	8	176.357	1.159	48.74
Within populations	156	190.225	1.219	51.26
Total	164	366.582	2.379	

Note

Significance tests (1,023 permutations); *p* < .001.

**FIGURE 3:**
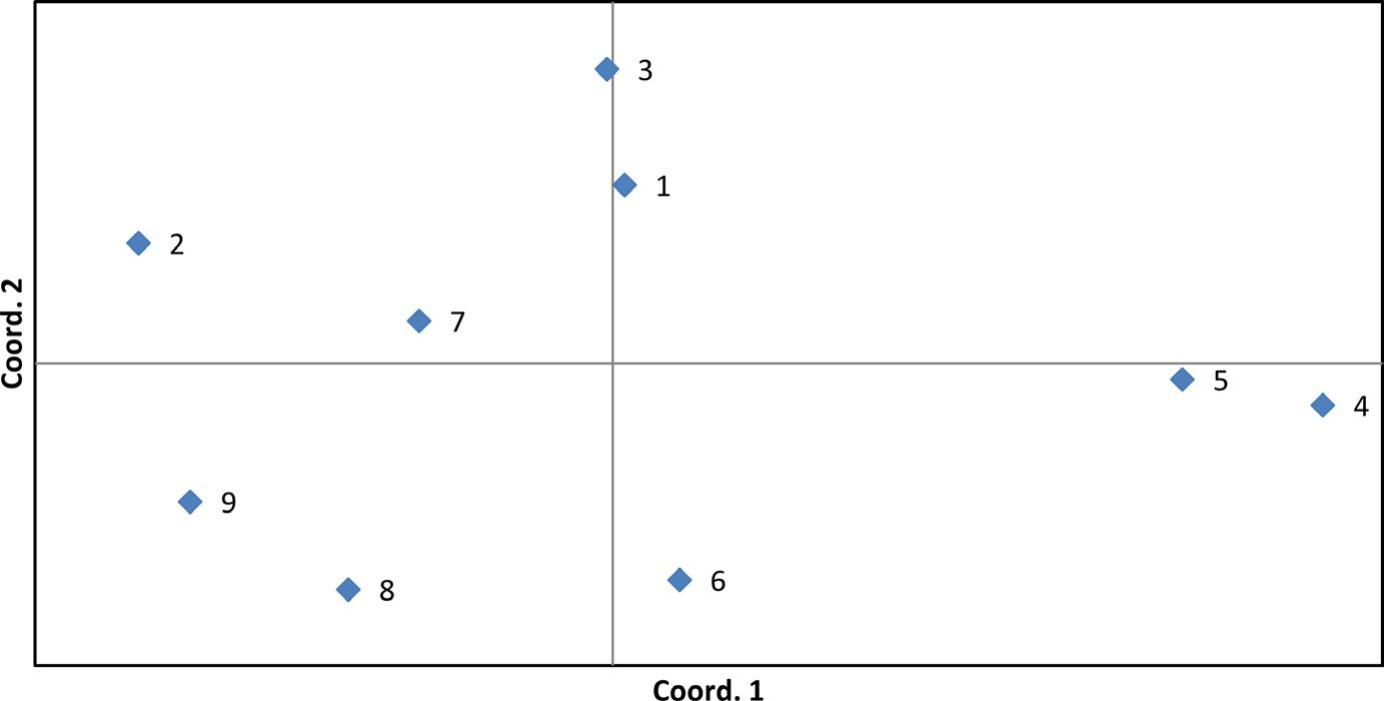
Projection of studied *Deschampsia antarctica* populations on the first two axes after principal coordinates analysis based on Nei's genetic distance values. Numbers of populations according to Table 1

Nei's genetic distance was calculated to estimate genetic differentiation between *D. antarctica* populations (Table 5). This parameter ranged from 0.007 to 0.047 (0.021 on average). The results of PCoA based on D_N_ values demonstrated that 81.75% of variation was explained by the first three components (48%, 23%, and 10%, respectively). The projection of the analyzed populations on the first two axes is shown in Figure 3. The groups identified in PCoA revealed a pattern of interpopulation genetic variation, where the analyzed populations were scattered along both axes. Two pairs of populations shared the highest degree of similarity: populations 4 and 5 which diverged along the first axis, and populations 1 and 3 which diverged along the second axis; populations 2, 8, and 9 had a peripheral position, population 7 was located somewhere in the middle of mentioned above groups of populations, whereas population 6 diverged from the other populations along the Coord.2.

**TABLE 5:** Nei's genetic distance values between studied *Deschampsia antarctica* populations. Numbers of populations according to Table 1

	1	2	3	4	5	6	7	8	9
1	^***^								
2	0.015	^***^							
3	0.009	0.016	^***^						
4	0.026	0.047	0.028	^***^					
5	0.015	0.040	0.021	0.004	^***^				
6	0.017	0.026	0.026	0.025	0.017	^***^			
7	0.011	0.018	0.013	0.036	0.022	0.018	^***^		
8	0.022	0.016	0.026	0.035	0.027	0.013	0.012	^***^	
9	0.018	0.011	0.025	0.043	0.034	0.016	0.012	0.007	^***^

^***^ refers to 0.0 Nei's genetic distance.

**FIGURE 4:**
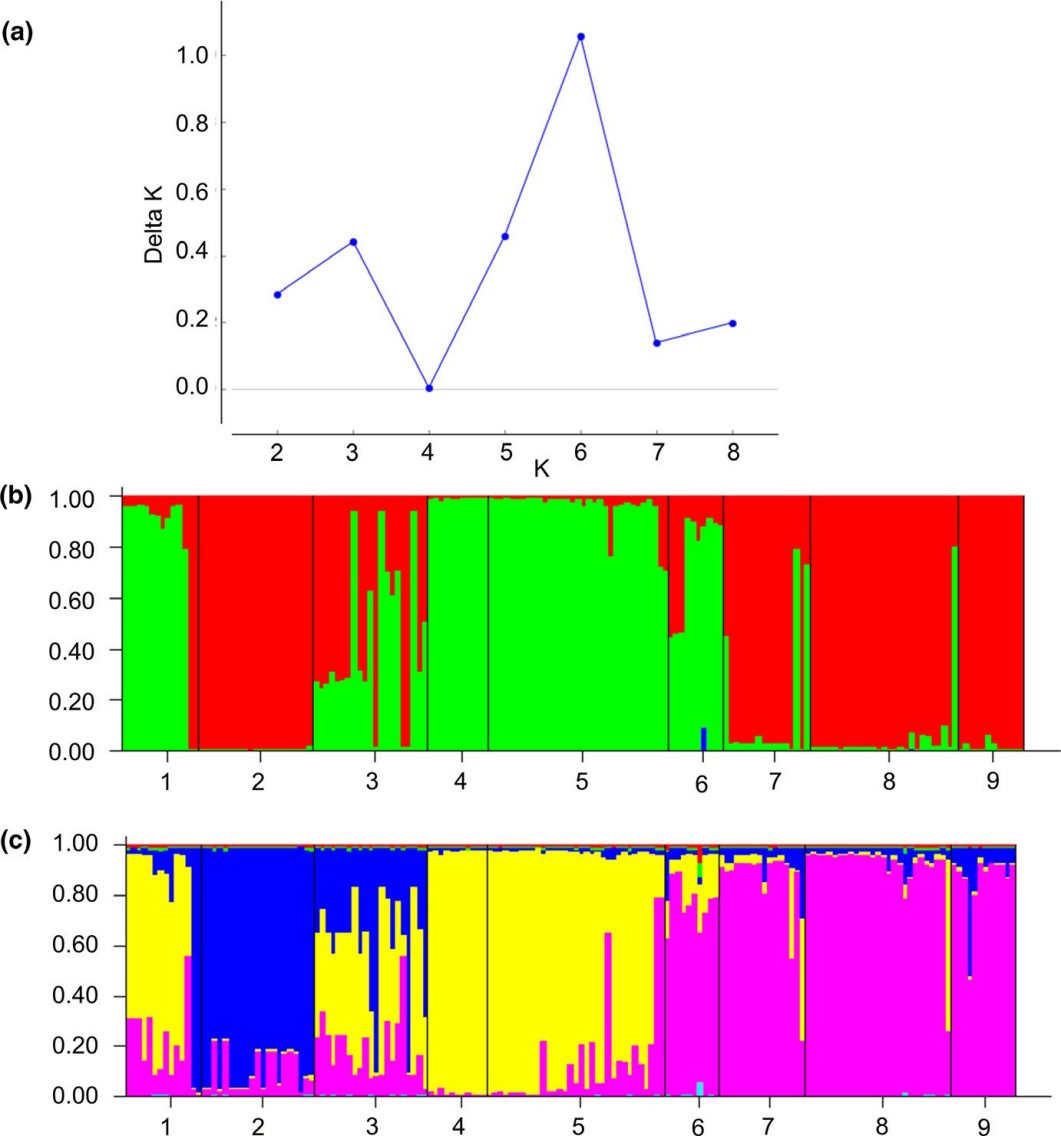
Structure of *D. antarctica* populations revealed by Bayesian analysis implemented in Structure. (a) Change (delta K) in likelihood for *K* = 1–9 (Evanno et al., 2005). (b) Individual probability assignment of each of the individuals sampled from 9 populations for *K* = 3. (c) Individual probability assignment of each of the individuals sampled from 9 populations for *K* = 6. Each individual is represented by a vertical bar broken into different colored genetic clusters, with length proportional to probability of assignment to each cluster

In order to infer about the genetic structure of the studied *D. antarctica* populations Bayesian model‐based clustering method was also applied, and the optimal number of clusters was estimated with the Δ*K* method (Evanno et al., 2005) The Δ*K* produced the highest peak at *K* = 6 with a minor peak at *K* = 3 (Figure 4a). When *K* = 3, populations 2, 8, and 9 were assigned to one cluster, populations 4 and 5 to the second one, whereas the other populations appeared to be populations with a certain degree of substructure between the two. Additionally, population 6 (due to additional, unique admixture) was characterized by the highest proportion of membership of each predefined population in the third cluster (Figure 4b). When *K* = 6, populations 1 and 3 appeared to be the most admixed populations; moreover, most populations showed population substructure, with population 4 standing out as nearly homogenous population (Figure 4c). Although the highest peak of Δ*K* point at *K* = 6 as the most likely number of clusters, bar plot for each individual genotypes for *K* = 3, sorted by population, is somewhat similar in composition to the population grouping based on PCoA.

#### 3.2.1 The effects of geographic and environmental isolation on population differentiation

**FIGURE 5:**
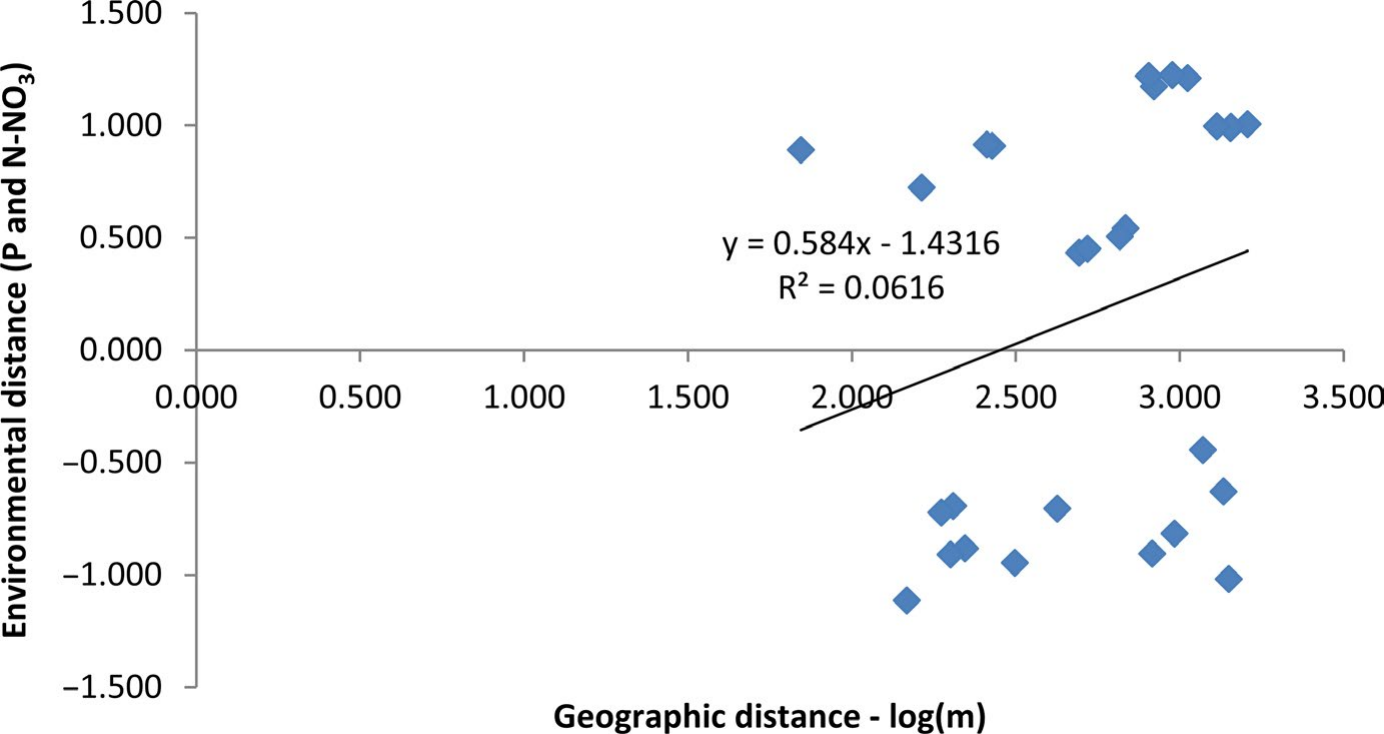
IBE analysis. The Mantel test scatterplot shows environmental distance (standardized to mean of zero and a standard deviation of one) as a function of logarithm of geographic distance

Rousset's isolation by distance method did not reveal any correlations between genetic and geographic pairwise distances (*R* ^2^ = 0.044; *p* = .08), which suggests that geographic distance did not influence genetic structure. The IBE Mantel test comparing geographic distance with pairwise environmental distance (calculated based on combined data for 12 soil properties and 10 variables describing nutrient content) did not reveal significant correlations, either. However, when environmental variables were analyzed separately, a significant correlation was found between geographic distance versus. the content of P and N–NO_3_ (*R* ^2^ = 0.0616, *p* = .041) (Figure 5).

#### 3.2.2 Neutrality tests and demography

Tajima's *D* did not reveal any deviations from 0, while Fu's *F*_S_ was negative and significant for most populations excluding population 2, 6, and 9 (Table 6), for which *p*‐value was over 0.02. As it was described by Fu (1997), *F*_S_ statistic should be considered as significant at the 5% level, if its *p*‐value is below .02, and not below .05. In the mismatch distribution test for demographic/spatial expansion, SSD values were not significant, and all samples had a very low raggedness index (Table 7).

**TABLE 6:** Tajima's *D* test and Fu's *F*_S_ neutrality tests of nine populations of *Deschampsia antarctica*

Test	Description	Populations									Statistics	
		1	2	3	4	5	6	7	8	9	Mean	*SD*
Tajima's *D* test	S	8	6	7	7	9	6	7	7	6	7	1
	*P_i_*	2.593	1.981	2.591	2.327	3.144	2.289	2.408	2.245	1.485	2.340	0.454
	Tajima's *D*	0.117	0.590	1.074	−0.106	1.271	0.328	0.499	0.716	−0.964	0.392	0.667
	Tajima's *D* *p*‐value	0.592	0.740	0.873	0.503	0.917	0.657	0.707	0.793	0.174	0.662	0.224
Fu's *F*_S_ test	Theta *p_i_*	2.593	1.981	2.590	2.327	3.144	2.289	2.408	2.245	1.485	2.340	0.454
	Exp. no. of alleles	5.266	5.353	6.199	4.508	8.160	4.288	5.366	6.261	3.774	5.464	1.306
	*F*_S_	−5.282	−3.194	−5.771	−8.151	−15.509	−2.845	−6.452	−8.210	−2.145	−6.395	4.062
	*F*_S_ *p*‐value	0.001	0.022	0.004	0.000	0.000	0.032	0.001	0.000	0.027	0.010	0.013

Note

*S*, number of segregating sites; *P_i_*, mean number of pairwise differences; Theta *p_i_*, Watterson's theta based on S; *F*_S_, Fu's *F*_S_; *SD*, standard deviation.

**TABLE 7:** Estimates of mismatch analysis of nine populations of *Deschampsia antarctica*

Model	Statistic	1	2	3	4	5	6	7	8	9	Mean	*SD*
Demographic expansion	SSD	0.048	0.011	0.003	0.027	0.006	0.009	0.008	0.002	0.017	0.015	0.015
	Model (SSD) *p*‐value	0.067	0.361	0.631	0.228	0.192	0.717	0.446	0.883	0.421	0.439	0.265
	Raggedness index	0.086	0.054	0.032	0.129	0.049	0.070	0.066	0.014	0.140	0.071	0.042
	Raggedness *p*‐value	0.430	0.560	0.761	0.190	0.136	0.575	0.373	0.995	0.284	0.478	0.277
Spatial expansion	SSD	0.020	0.008	0.003	0.027	0.006	0.009	0.008	0.004	0.017	0.011	0.008
	Model (SSD) *p*‐value	0.220	0.540	0.606	0.171	0.157	0.623	0.354	0.797	0.343	0.423	0.227
	Raggedness index	0.086	0.054	0.032	0.129	0.049	0.070	0.066	0.014	0.140	0.071	0.042
	Raggedness *p*‐value	0.298	0.630	0.730	0.190	0.126	0.533	0.345	0.998	0.275	0.458	0.286

Abbreviations: *SD*, standard deviation; SSD, sum of squared deviations; Raggedness index, Harpending's raggedness index.

#### 3.2.3 The mutation−drift model versus the bottleneck hypothesis

All three tests used in this study analyze bottleneck effects in populations that develop transient heterozygosity excess. If the loci evolve in a strict one‐step mutation model, heterozygosity excess and deficiency can occur depending on locus variability and the time elapsed since the beginning of the bottleneck. In three of the analyzed populations (1, 3, and 5), significant traces of bottleneck effect were noted in populations 1 and 5 based on the results of all three tests (Sign test, Standardized Differences test, and Wilcoxon test), and in population 3 based on the results of the Standardized Differences test and the Wilcoxon test, but the *p*‐value in the Sign test was very close to the significance level (Table 8).

**TABLE 8:** Testing bottleneck versus mutation–drift equilibrium hypotheses for all analyzed populations (IAM mutation model)

Population	SIGN Test	Standardized test	Wilcoxon test
1	Hee = 3.19 Hd = 0 He = 7 *p* = .00409	T2 = 3.058 *p* = .00112	One tail for heterozygosity deficiency: 1.00000
			One tail for heterozygosity excess: 0.00391
			Two tails for heterozygosity excess and deficiency: 0.00781
2	Hee = 2.09 Hd = 2 He = 3 *p* = .34933	T2 = 0.343 *p* = .36589	One tail for heterozygosity deficiency: 0.59375
			One tail for heterozygosity excess: 0.5000
			Two tails for heterozygosity excess and deficiency: 1.00000
3	Hee = 2.56 Hd = 1 He = 5 *p* = .05504	T2 = 1.741 *p* = .04081	One tail for heterozygosity deficiency: 0.97656
			One tail for heterozygosity excess: 0.03906
			Two tails for heterozygosity excess and deficiency: 0.07813
4	Hee = 2.39 Hd = 3 He = 3 *p* = .45257	T2=−0.216 *p* = .41430	One tail for heterozygosity deficiency: 0.50000
			One tail for heterozygosity excess: 0.57813
			Two tails for heterozygosity excess and deficiency: 1.0000
5	Hee = 3.45 Hd = 1 He = 7 *p* = .01388	T2 = 3.273 *p* = .00053	One tail for heterozygosity deficiency: 0.99805
			One tail for heterozygosity excess: 0.00391
			Two tails for heterozygosity excess and deficiency: 0.00781
6	Hee = 2.74 Hd = 1 He = 4 *p* = .25066	T2 = 0.939 *p* = .17374	One tail for heterozygosity deficiency: 0.92188
			One tail for heterozygosity excess: 0.10938
			Two tails for heterozygosity excess and deficiency: 0.21875
7	Hee = 2.92 Hd = 2 He = 4 *p* = .31898	T2 = 0.608 *p* = .27153	One tail for heterozygosity deficiency: 0.71875
			One tail for heterozygosity excess: 0.34375
			Two tails for heterozygosity excess and deficiency: 0.68750
8	Hee = 2.85 Hd = 2 He = 4 *p* = .29867	T2 = 1.107 *p* = .13412	One tail for heterozygosity deficiency: 0.94531
			One tail for heterozygosity excess: 0.07813
			Two tails for heterozygosity excess and deficiency: 0.15625
9	Hee = 2.08 Hd = 2 He = 3 *p* = .34541	T2 = 0.269 *p* = .39377	One tail for heterozygosity deficiency: 0.68750
			One tail for heterozygosity excess: 0.40625
			Two tails for heterozygosity excess and deficiency: 0.81250

Abbreviatins: Hee, expected heterozygosity excess; Hd, heterozygosity deficiency; He, heterozygosity excess.

## 4 DISCUSSION

Antarctic hairgrass is one of the most intriguing plant species in the world whose unique morphological and physiological features enable survival in extreme environments and colonization of the remote and inhospitable areas of the Maritime Antarctic. The species has large disjunctive distribution from north Patagonia in South America (around 38°S) to the Antarctic Peninsula, with the southernmost known locality in Lazer Bay (Alexander Island, 69°22.0’S, 71°50.7’W; Convey, 2011). Extensive geographic distribution has contributed to high morphological and anatomical variation of the species (Chwedorzewska et al., 2008; Giełwanowska & Szczuka, 2005; Giełwanowska et al., 2005). However, morphological plasticity is not accompanied by equally extensive genetic variability, which is generally low in the entire species range. A discrete decrease in genetic diversity is observed from the north to the south, with minimum values in the area of the Antarctic Peninsula (Holderegger et al., 2003; Chwedorzewska & Bednarek, 2008; van de Wouw et al., 2008).

According to the literature, *D. antarctica* colonized Antarctica during the Holocene (Chapman, 1996; Lewis‐Smith, 1984). In the paleobotanical research conducted by Birkenmajer et al. (1985), the fragments and pollen of *D. antarctica* isolated from peat cores were dated back at least five millennia. Reliable conclusions about the evolutionary history of Antarctic lichens, bryophytes, and flowering plants are difficult to draw due to insufficient data on contemporary species distribution. Recent molecular phylogeographic studies and classical biogeographic studies provided strong evidence that the persistence of Antarctica's extant terrestrial biota spans hundreds of thousands to millions of years (Convey, Bindschadler et al., 2009; De Wever et al., 2009; Domaschke et al., 2013; Fraser et al., 2014; McGaughran et al., 2010; Pisa et al., 2014; Romeike et al., 2002; Vyverman et al., 2010; Chong et al. 2015). The hypothesis postulating the presence of Antarctic glacial refugia during the Pleistocene was recently supported by glaciological evidence and population genetics data from various groups of organisms (Convey, Stevens et al., 2009; De Wever et al., 2009; McGaughran et al., 2010). For example, a recent population genetics study of the cosmopolitan moss *Bryum argenteum* Hedw. suggested its long‐term persistence in the Antarctic, which was reflected in its low genetic diversity (Clarke et al., 2009; Hills et al., 2010; Pisa et al., 2014).

To date, most molecular studies of *D. antarctica* have relied on the AFLP technique AFLP data (Chwedorzewska et al., 2004, 2008) demonstrated that *D. antarctica* populations which originated in close vicinity share considerable genetic similarity, but have evident morphological and anatomical differences. In the cited studies, the total percentage of polymorphic loci in the AFLP analysis did not exceed 39%. In studies that covered a wider geographic range of the species, polymorphism varied significantly from 13% (in ten populations from Signy Island, Anchorage Island, Lagoon Island, and Léonie Island in northern and southern Maritime Antarctic, respectively; Holderegger et al., 2003) to 92% within 38 *D. antarctica* populations from the sub‐Antarctic islands in the Indian Ocean, the Falklands, South Georgia Island, and the Antarctic Peninsula with the adjacent islands (van de Wouw et al., 2008). The high polymorphism demonstrated by AFLP was not confirmenoncoding regions of chloroplast genomesd by analyses of genetic variation within selected (van de Wouw et al., 2008).

In this study, iPBS markers supported the identification of 15 (12%) polymorphic loci, and average polymorphism was determined at 5.6% (across loci and populations). Previous studies relied on iPBS markers demonstrated up to 97% polymorphism in *Myrica rubra* (Chen & Liu, 2014). Lower polymorphism for iPBS markers (5%) was reported only by Baránek et al. (2012), but their study aimed to identify the clones of an apricot cultivar.

iPBS markers were previously applied to investigate the genetic diversity of Antarctic pearlwort (*Colobanthus quitensis*) (Androsiuk et al., 2015). The genotyping of individuals from eight *C. quitensis* populations in the vicinity of Henryk Arctowski Station revealed higher polymorphism than in *D. antarctica,* where 55 of 143 loci (39%) were polymorphic, with 14% of polymorphic loci per population on average. The average values of *H*_e_ and *I* were determined at 0.040 and 0.061, respectively, which indicates that genetic diversity in *C. quitensis* was twice higher than that noted in *D. antarctica* in this study (average values of uH_e_ = 0.021, *I* = 0.030). The results of AMOVA revealed 84% of the polymorphisms within the studied populations of *C. quitensis* and only 51% within populations of *D. antarctica*. As a result, the hierarchical analysis of population structure produced clearly higher values of the *F*‐statistic for *D. antarctica* (*F*_ST_ = 0.4874) than *C. quitensis* (*F*_ST_ = 0.164). Our results are highly consistent with the observations made by van de Wouw et al. (2008), in whose study, AMOVA revealed 46% of total genetic diversity among *D. antarctica* populations from Antarctic sites. Holderegger et al. (2003) also reported that 45% of genetic variation in *D. antarctica* from the Southern Maritime Antarctic was partitioned between populations. Unsurprisingly, genetic diversity among populations clearly decreased when a wider geographic range was analyzed, whereas the variation among regions increased to 37% (northern versus southern Maritime Antarctic; Holderegger et al., 2003) or even 75% when *D. antarctica* from the sub‐Antarctic islands in the Indian Ocean, the Falklands, South Georgia Island, and the Antarctic Peninsula with the adjacent islands were considered (van de Wouw et al., 2008).

*Deschampsia antarctica* is characterized by wide ecotypic variation (Lewis‐Smith, 2003; Nędzarek & Chwedorzewska, 2004); however, the genetic variation associated with the observed phenotypic dissimilarities has not been elucidated to date (van Fasanella et al., 2017; de Wouw et al., 2008). Polar plants have developed a number of response mechanisms to various biotic and abiotic stresses (Bruce et al., 2007; Cui et al., 2020; Giełwanowska et al., 2015). Some of these mechanisms can contribute to phenotypic variation caused by mutation (Rout et al., 2006) or modification of the DNA methylation pattern (Chinnusamy & Zhu, 2009). Chwedorzewska and Bednarek (2011), using methylation sensitive AFLP approach (metAFLP platform), found that methylation played a crucial role in the phenotypic variation of the *D. antarctica* specimens from different habitats of King George Island. Inconspicuous polymorphisms in the methylation pattern that have emerged in response to various stresses may be crucial in acclimatization to a range of environmental conditions, and they could be responsible for the differentiation of particular populations into local ecotypes (Cubas et al., 1999; Stajic & Bank, 2020).

A different approach was used in our previous study (Androsiuk et al., 2015), where the applicability of retrotransposon‐based molecular markers (iPBS technique) was verified in an analysis of genetic variation in *C. quitensis*. Similarly to other TEs, retrotransposons are mobilized in response to various stress factors (Capy et al., 2000; Schrader et al., 2014). Despite their random nature, genome rearrangements caused by TE activation could be beneficial because newly arisen genetic variation may be associated with adaptation to certain abiotic stressors (Finatto et al., 2015). A study of *Hordeum spontaneum* from the Evolution Canyon microsite in Lower Nahal Oren, Mount Carmel in Israel provided highly valuable insights (Kalendar et al., 2000). The authors reported an increase in the activity of *BARE*‐1 retrotransposons in *H. spontaneum* individuals growing on a slope exposed to high temperatures and drought. Retrotransposon‐based polymorphism allowed the identification of individuals that were and were not stressed by drought, even though the two sites were separated by a distance of only 300 m. The authors observed that local data were consistent with *BARE*‐1 trends in *H. spontaneum* throughout Israel and, therefore, could reflect adaptive selection for increasing genome size through retrotransposon activity (Kalendar et al., 2000). Similar observations were made in our previous study, where the genetic polymorphism analysis of *C. quitensis* revealed that TEs could be mobilized in response to various abiotic stressors (Androsiuk et al., 2015).

The results of the present study clearly indicate that despite low genetic polymorphism assessed with iPBS markers (6% on average) and low genetic diversity (*H*_e_ = 0.020), the analyzed populations of *D. antarctica* are characterized by relatively high genetic differentiation (*F*_ST_ = 0.4874, *G*_st_‐B = 0.4349, *θ* ^(^ *^III^* ^)^ = 0.3703, results of STRUCTURE analysis which assigned the studied individuals into 6 clusters, and high population dispersal revealed by PCoA), which could be attributed to limited gene transfer between these populations. This is a surprising observation in view of the fact that the analyzed populations were sampled in close proximity. However, local differences in landform and climatic conditions, such as strong winds blowing mainly from one direction (Wierzbicki, 2009), could be responsible for hampering seed dispersal and seedling establishment. These observations suggest that gene flow was obstructed by environmental heterogeneity or circulation boundaries in the studied area rather than the physical distance between sampling sites (no evidence for IBD was found).

The observed phenomena could also be attributed to the unique characteristics of the study area. The Maritime Antarctic is an extraordinary region not only due to extreme environmental conditions, but also their dynamics and diversity. Plants which colonized the studied area had to adapt to the local mosaic of microhabitats as well as general climatic fluctuations (Convey, 1996). Therefore, each site is characterized by dozens of factors describing microclimate conditions and soil properties that may vary considerably even between closely located sites. In extreme cases, even parts of the same population can experience different environmental conditions (Lachacz et al., 2018). For this reason, the genetic polymorphisms in the studied *D. antarctica* populations could have been shaped independently due to random TE mobilization and the spatial distribution of populations in an area with diverse abiotic stressors. The results of the IBE analysis revealed that environmental heterogeneity could have influenced the observed genetic differentiation. Most notably, the presence of a significant correlation between P and N–NO_3_ content versus. *F*_ST_/(1−*F*_ST_) indicates that differences in nutrient content could be associated with population divergence. However, further tests are needed to fully determine whether the differences in the composition and nutrient content of soils in the Antarctic oasis (Lachacz et al., 2018) are responsible for promoting certain genotypes.

Cytological analysis revealed that the common chromosome counts in *D. antarctica* is 2*n* = 26 (Amosova et al., 2015; Cardone et al., 2009; Volkov et al., 2010), although some counts reported also polyploid populations of 2*n* = 52 in Patagonia, which has not been reported in Antarctic populations (e.g., González et al., 2016). It has been suggested that polyploidization is one of the main factors responsible for shaping diversity in angiosperms (Leitch & Leitch, 2008). According to some studies, polyploidization could result in higher level of polymorphism revealed by molecular markers (e.g., Budak et al., 2005; Gulsen et al., 2009; Milla‐Lewis et al., 2013). However, there are also other studies which show lack of significant influence of polyploidization on genetic variation (e.g., Liu et al., 2001; Zeng et al., 2011; Zhang et al., 2017). Research conducted in marine Antarctica has shown that diploid individuals of *D. antarctica* predominate in the populations of this area. However, especially in the Southernmost populations the hypotriploid individuals and genotypes that had the B chromosome were also reported (Amosova et al., 2015; Navrotska et al., 2017). The presence of mixoploid plants in *D. antarctica* populations is maintained by its capacity of vegetative and apomictic propagation common in populations from the range limit, where environmental stress conditions prevail (e.g., Amosova et al., 2015). However, as it was shown by Navrotska et al. (2017), genetic differentiation between *D. antarctica* individuals with anomalous karyotype and diploids did not differ significantly from genetic differentiation between individuals representing typical diploid plants. Therefore, more detailed cytological and molecular studies with extensive sampling are needed to check whether polyploidization in *D. antarctica* is common or rather rare event, and to test how the change in ploidy level may affect the genetic diversity of the species.

The reproductive biology of *D. antarctica* is yet another important factor which should be considered in evaluations of the evolution and maintenance of genetic diversity and differentiation in the species. *D. antarctica* is self‐compatible plant species which can reproduce by self‐pollinating cleistogamous flowers or by vegetative reproduction (Giełwanowska & Kellmann‐Sopyła, 2015; Parnikoza et al., 2018) which does not contribute to genetic variation. In favorable circumstances, the species can produce viable seeds by outcrossing (Convey, 1996). However, outcrossing is highly unlikely in the Maritime Antarctic due to adverse climate conditions which inhibit generative reproduction or prolong the development of viable seeds even to two growing seasons (Parnikoza et al., 2018). Very strong winds may also impede pollen and seed dispersal. Therefore, new genotypes have limited dispersal opportunities because the seeds of *D. antarctica* do not have any structures that could promote dispersal across long distances. The patchy nature of ice‐free areas that are often separated by impassable barriers such as glaciers or open sea waters can also limit gene flow. Parnikoza et al. (2008) reported that some Antarctic bird species (*Larus dominicanus*, *Catharacta maccormicki* and *C. lonnbergi*) use plants, to build nests, which suggests that zoochory could play a role in the local dispersal of *D. antarctica*.

Regardless of its dispersal mechanism, a newly established population often has a limited number of individuals, which reproduce mainly by self‐fertilization and/or vegetative propagation (Holderegger et al., 2003). In most cases, these individuals are the only source of genes for the successive generations. In the current study, the negative values of Fu's *F*_S_ statistic revealed a demographic expansion of most studied populations (except populations 2, 6 and 9), whereas significant traces of reduced effective population size were noted only in three populations (1, 3, and 5). The observed variations indicate that the studied *D. antarctica* populations had different demographic histories, which could be attributed to recurring adverse environmental conditions (that are different even for closely located, neighboring populations) or even recent local extinction–recolonization events. These processes could explain why bottlenecks were detected in only some of the analyzed populations. However, the difference between bottleneck and founder effects is difficult to identify with the use of dominant markers.

Our molecular data are consistent with the results of other studies on the genetic characteristic of *D. antarctica*. The present findings have confirmed the low genetic variation of *D. antarctica* and have demonstrated surprising differences between populations. The observed pattern of genetic differentiation probably reflects the local mosaic of microhabitats on King George Island. However, an analysis of the most southern range of the species indicates that genetic differentiation could also be attributed to specific landform which can isolate even close populations and limit generative propagation. Further research is needed to explore retrotransposon‐based polymorphism in greater detail and to confirm the association between abiotic stress factors and the polymorphisms revealed by iPBS markers.

## CONFLICT OF INTEREST

All authors declare no conflict of interest.

## AUTHOR CONTRIBUTION

**Piotr Androsiuk:** Conceptualization (equal); Data curation (lead); Formal analysis (lead); Investigation (equal); Methodology (equal); Resources (supporting); Supervision (lead); Validation (equal); Visualization (lead); Writing‐original draft (lead); Writing‐review & editing (lead). **Katarzyna J. Chwedorzewska:** Conceptualization (equal); Data curation (supporting); Formal analysis (supporting); Investigation (supporting); Methodology (equal); Resources (equal); Validation (equal); Visualization (supporting); Writing‐original draft (equal); Writing‐review & editing (equal). **Justyna Dulska:** Data curation (supporting); Investigation (equal); Writing‐review & editing (supporting). **Sylwia Milarska:** Data curation (supporting); Investigation (equal); Writing‐review & editing (supporting). **Irena Giełwanowska:** Conceptualization (equal); Data curation (supporting); Formal analysis (supporting); Investigation (supporting); Methodology (equal); Resources (equal); Supervision (equal); Validation (equal); Visualization (supporting); Writing‐original draft (equal); Writing‐review & editing (equal).

## Supporting information

Table S1

Table S2

## Data Availability

Binary matrix of iPBS data is available among Supplementary Materials (iPBS_data.xlsx) attached to this paper. Raw data (photographs of horizontal agarose gel electrophoresis) are available from the Dryad Digital Repository (https://doi.org/10.5061/dryad.cfxpnvx47).
