# Supplementary material for: Retrotransposon‐based genetic diversity of Deschampsia antarctica Desv. from King George Island (Maritime Antarctic)
Source: Ecol Evol. 2020 Dec 16;11(1):648–63. doi: 10.1002/ece3.7095 (PMC7790655; doi:10.1002/ece3.7095)

Supplementary File to:

Piotr Androsiuk, Katarzyna J. Chwedorzewska, Justyna Dulska, Sylwia Milarska and Irena Giełwanowska

Retrotransposon-based genetic diversity of *Deschampsia antarctica* Desv. from King George Island (Maritime Antarctic)

Table S1. Photographic documentation of nine *D. antarctica* populations from King George Island (photographs by Irena Giełwanowska). Numbers of populations according to Table 1.

Population #1

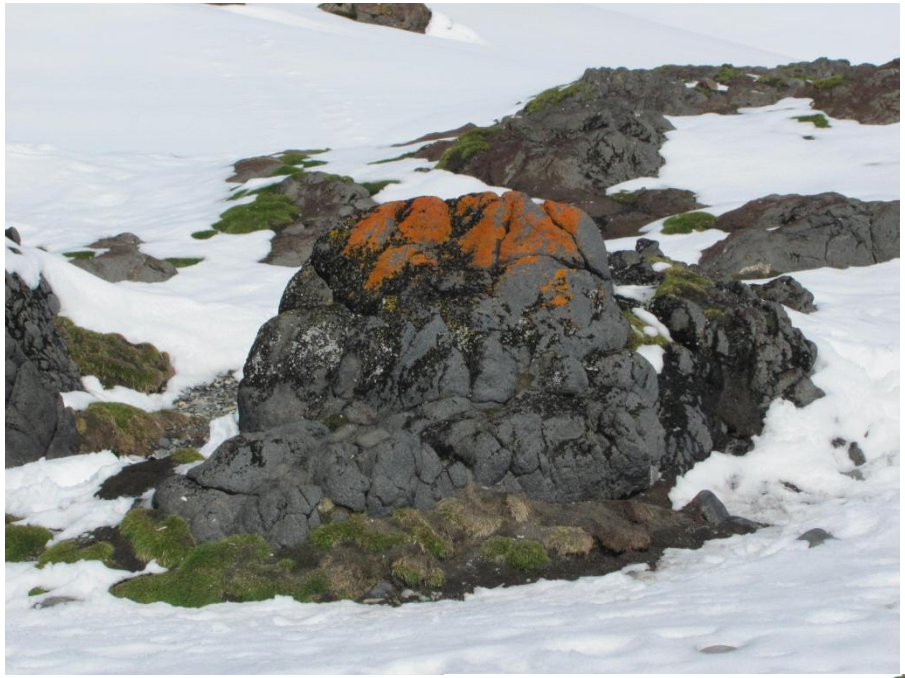

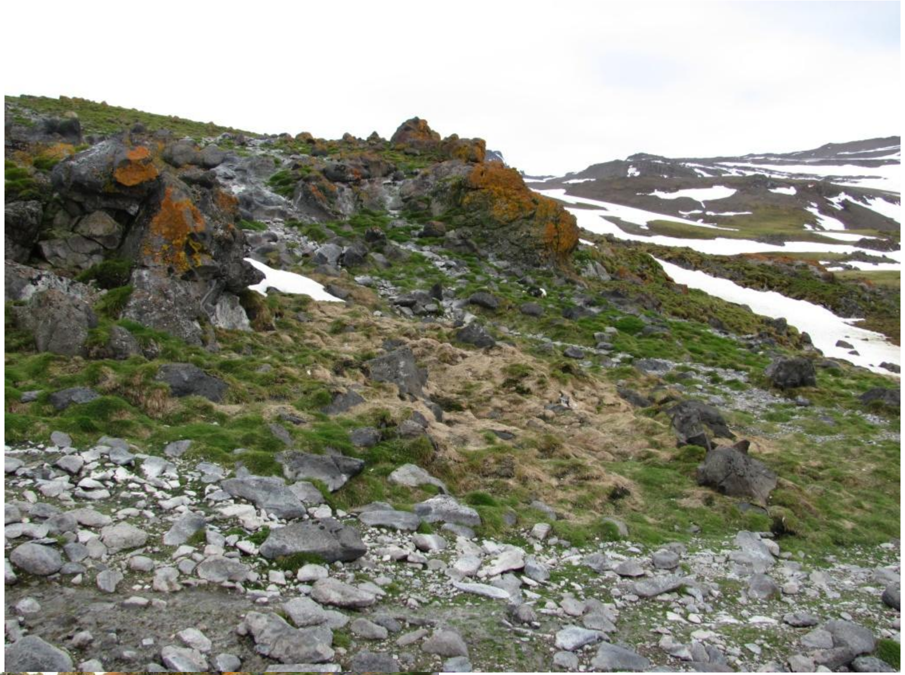

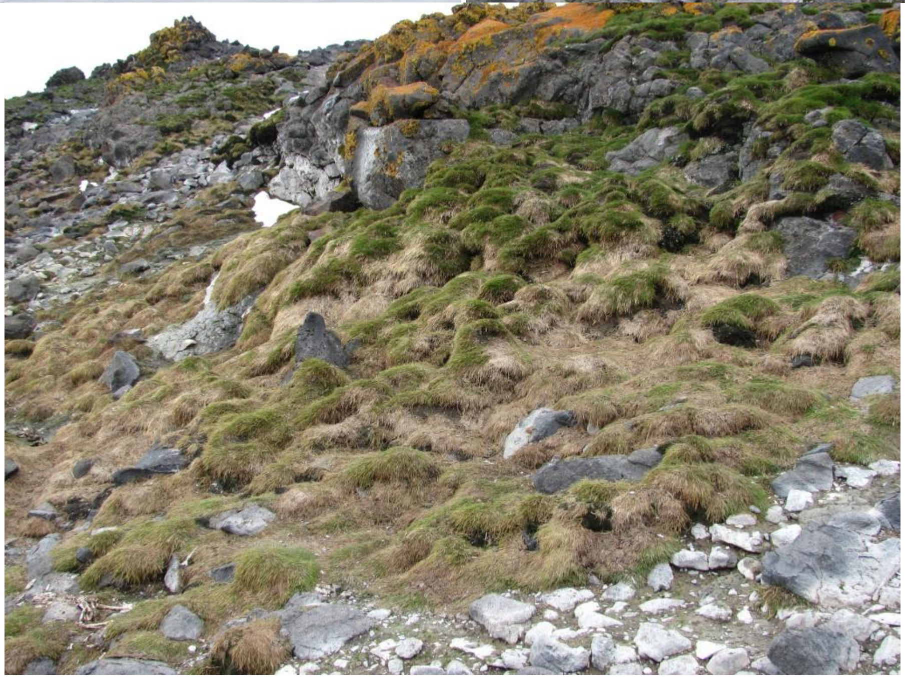

Population #2

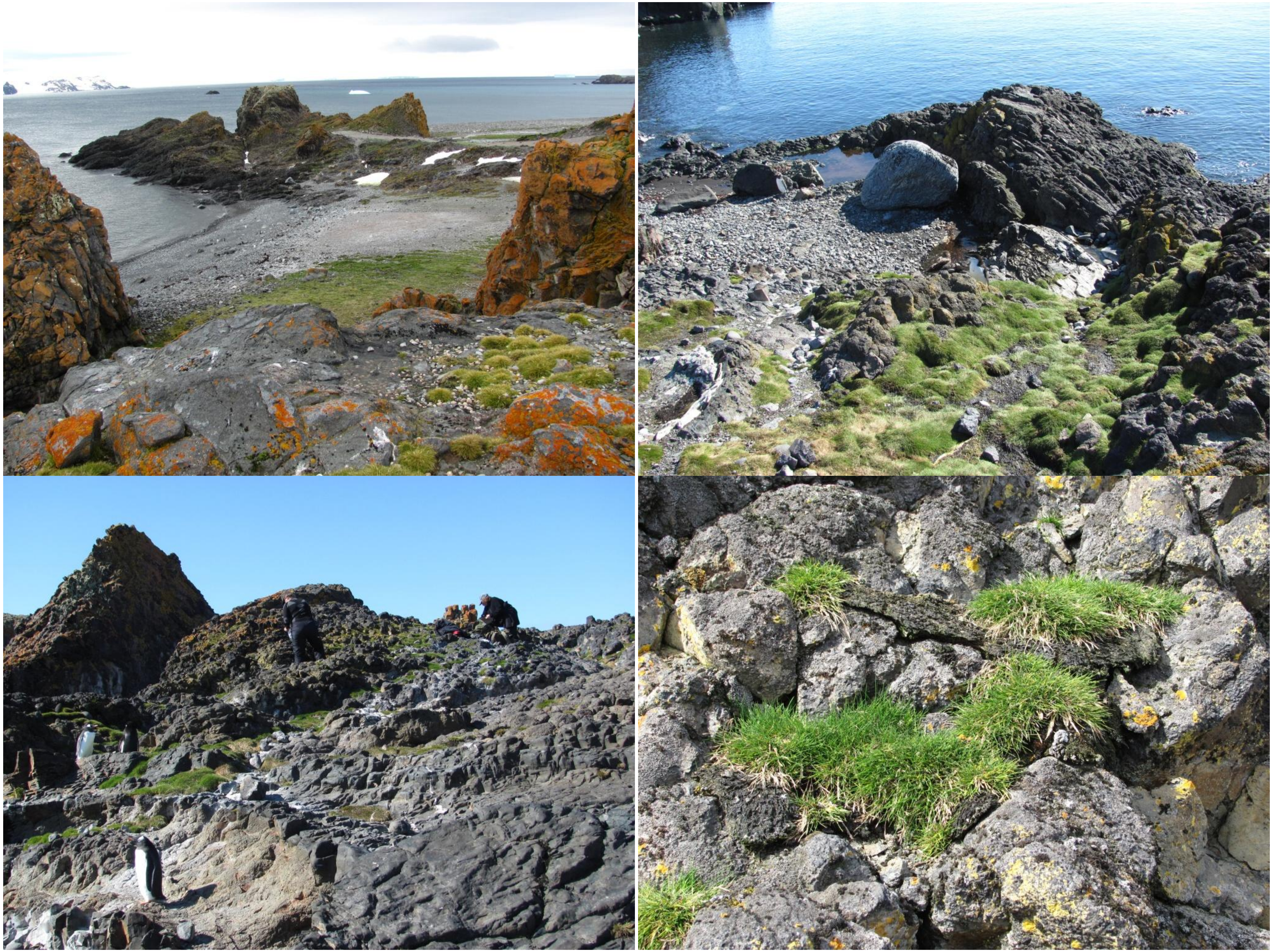

Population #3

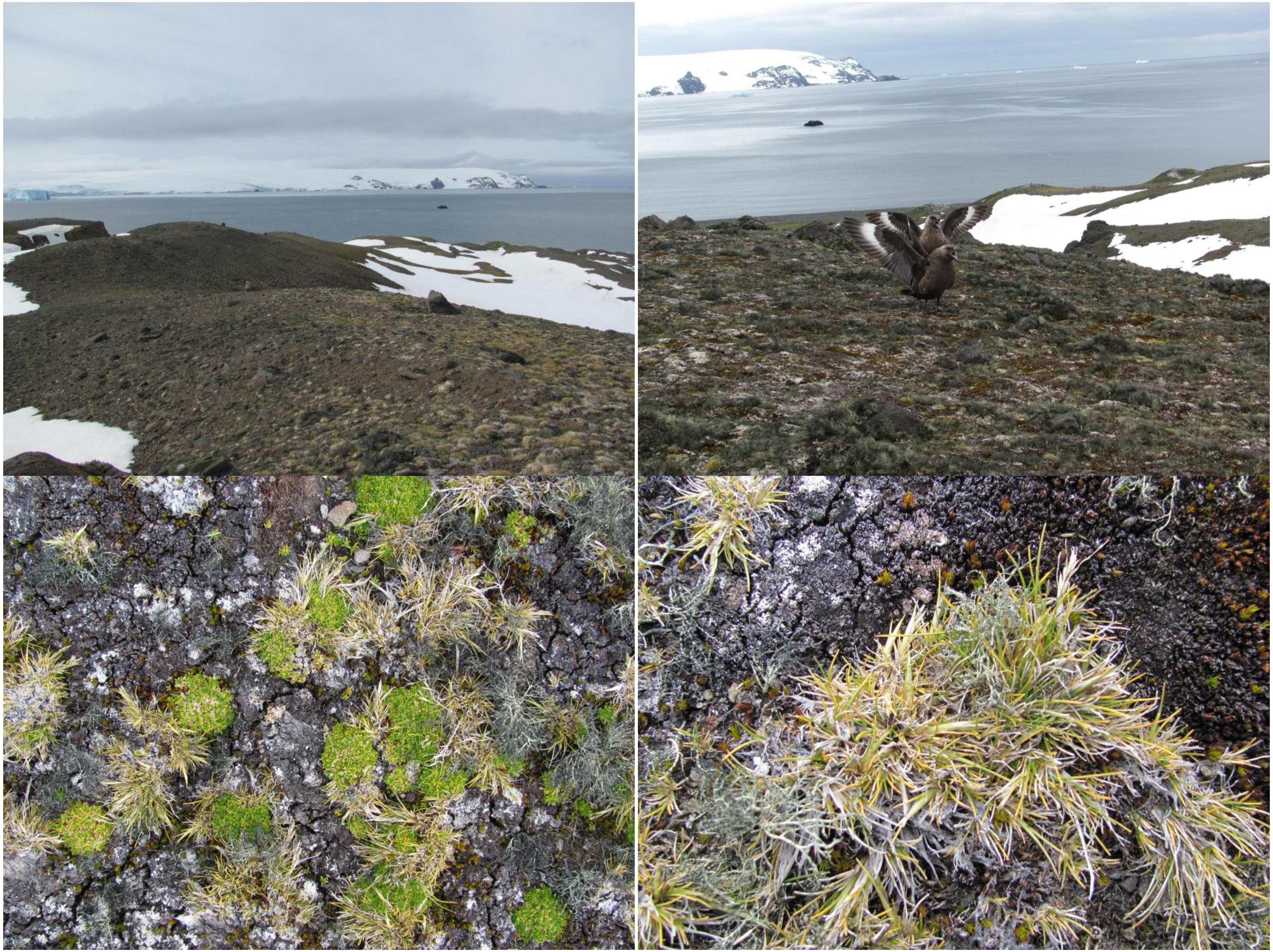

Population #4

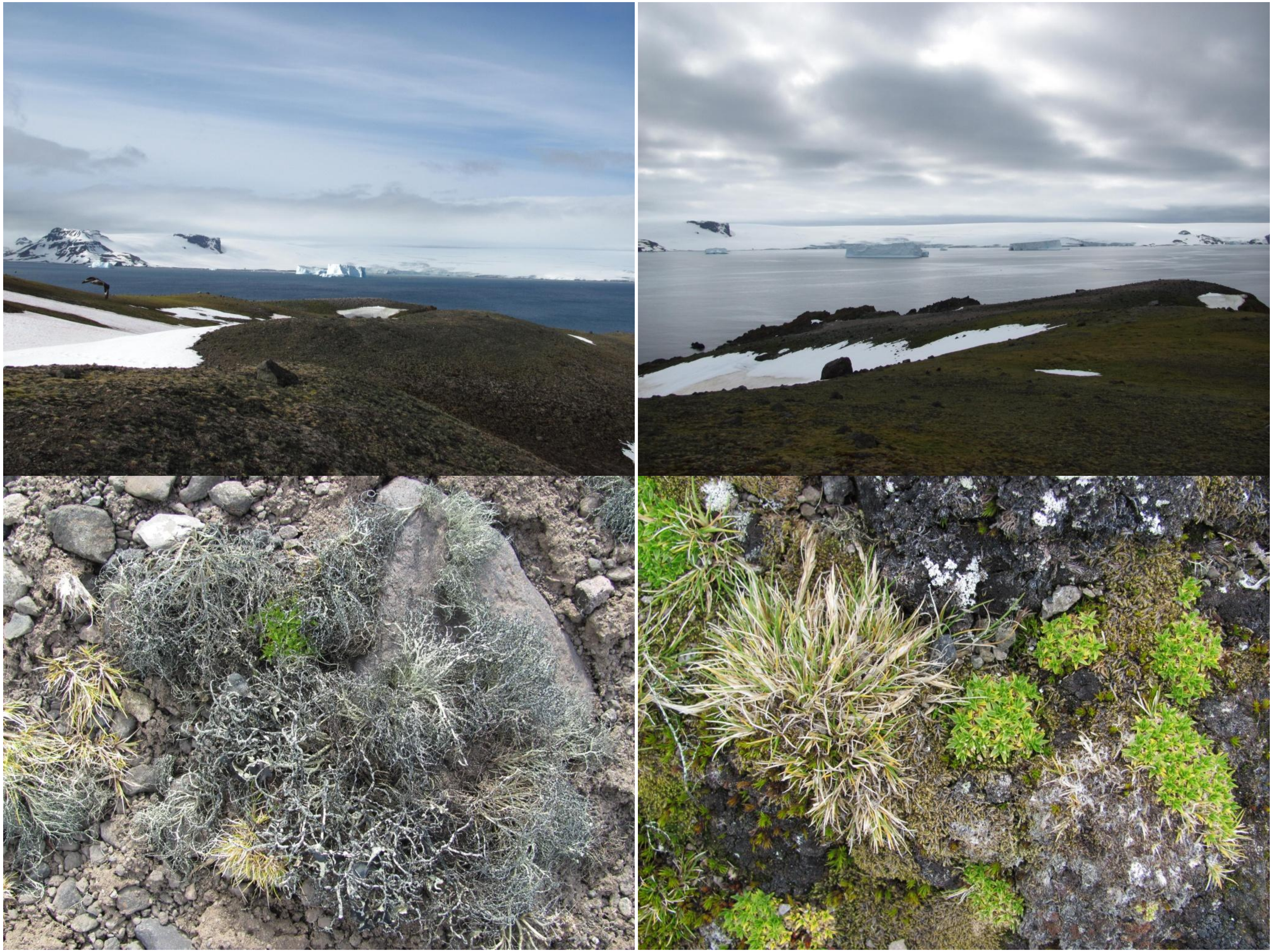

Population #5

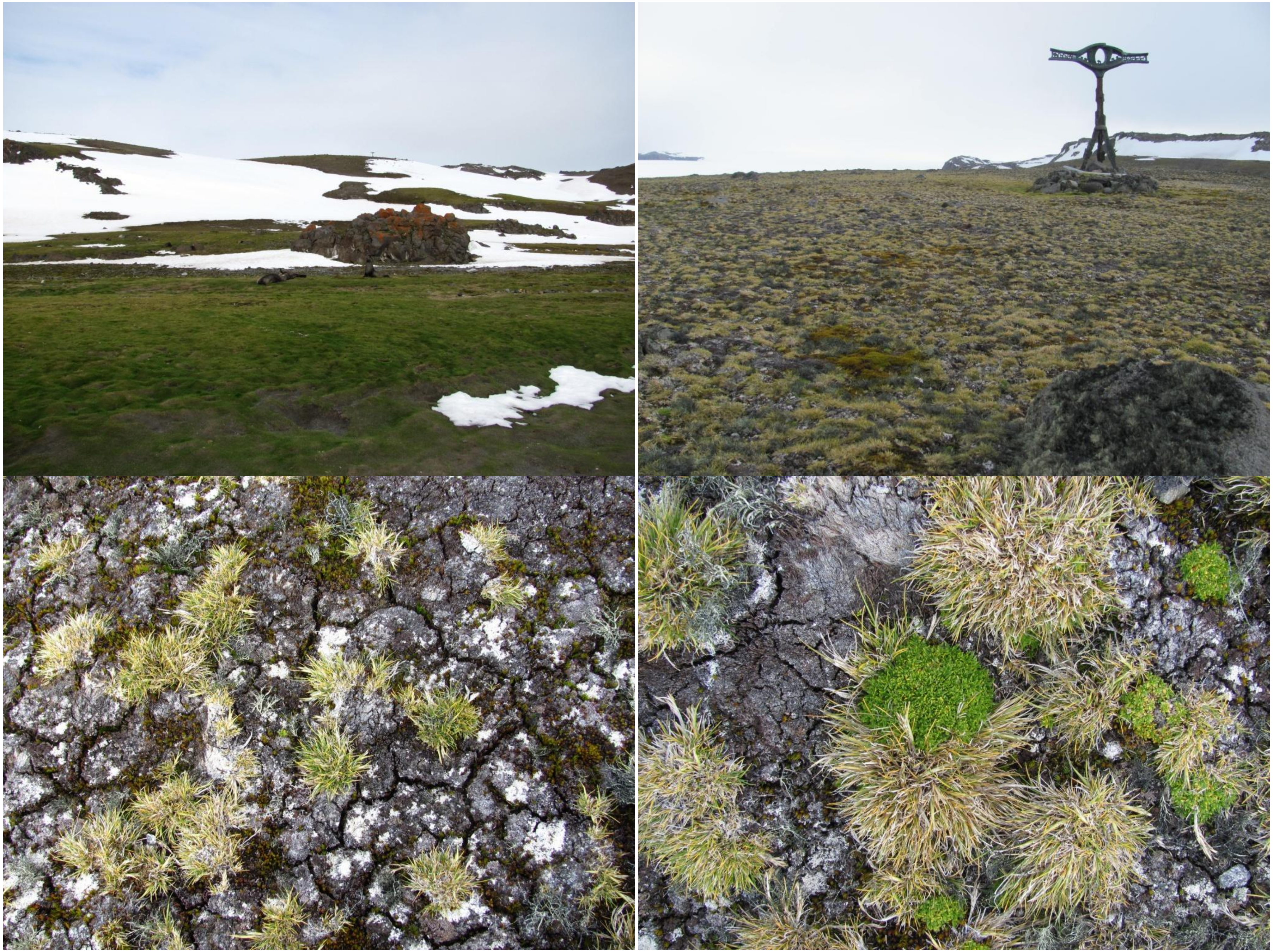

Population #6

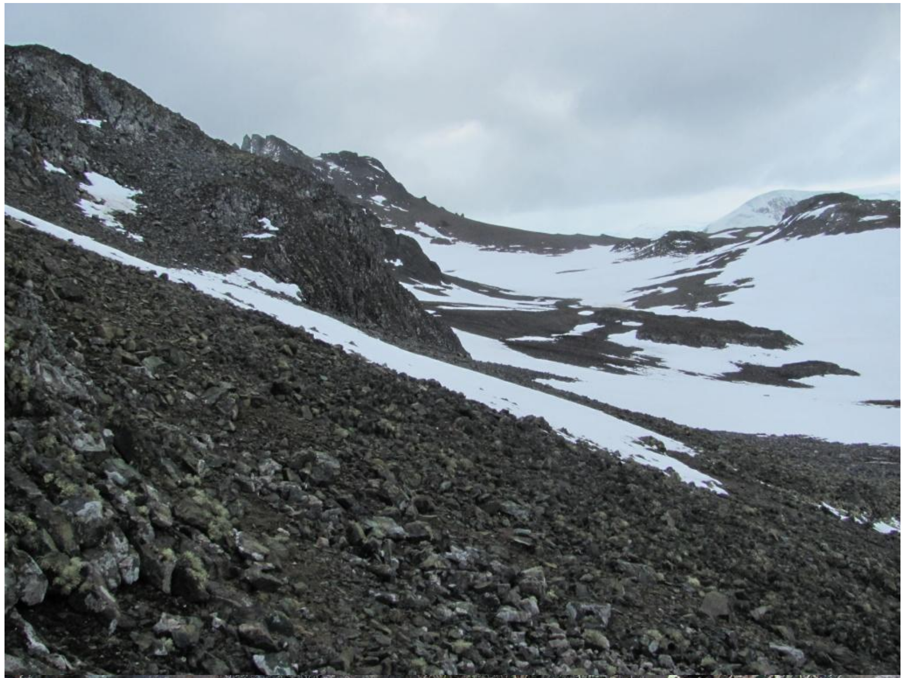

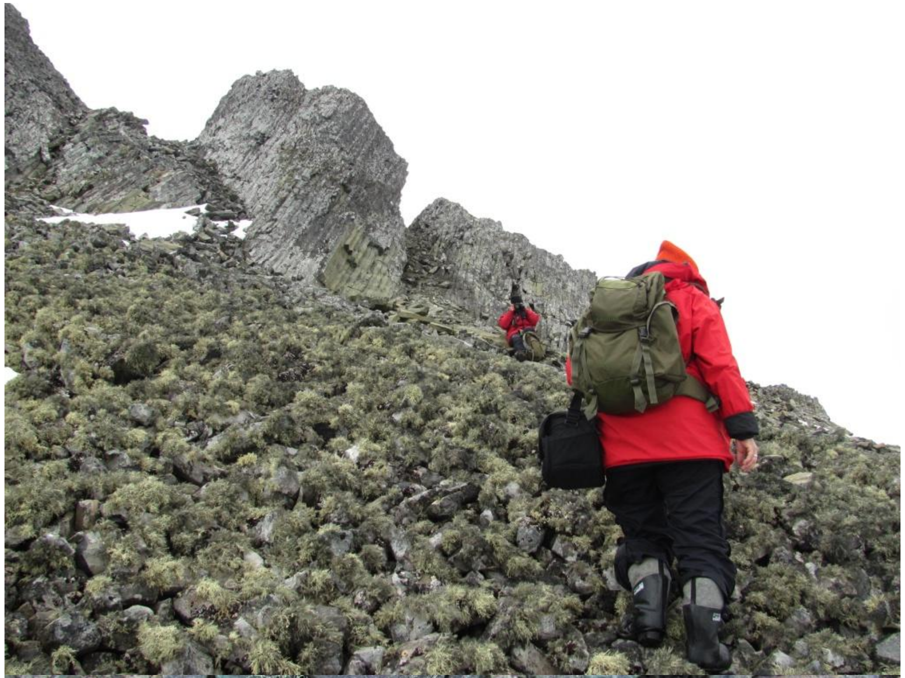

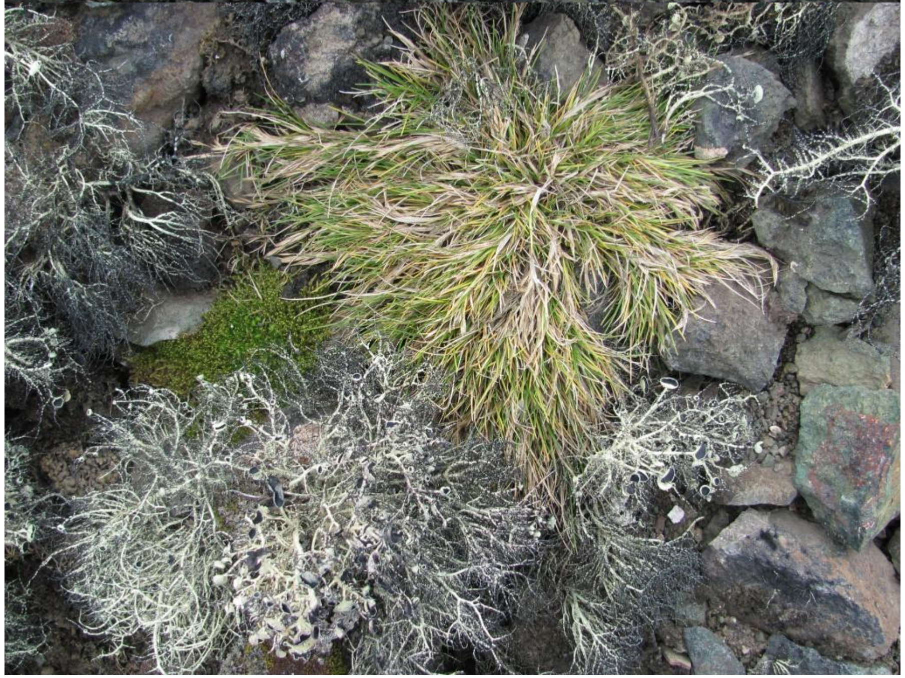

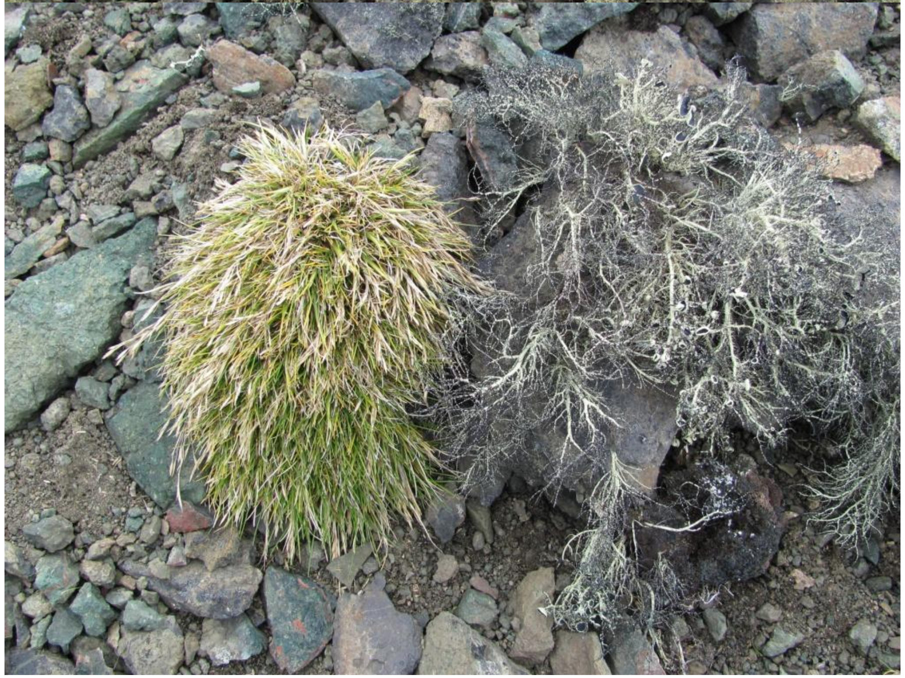

Population #7

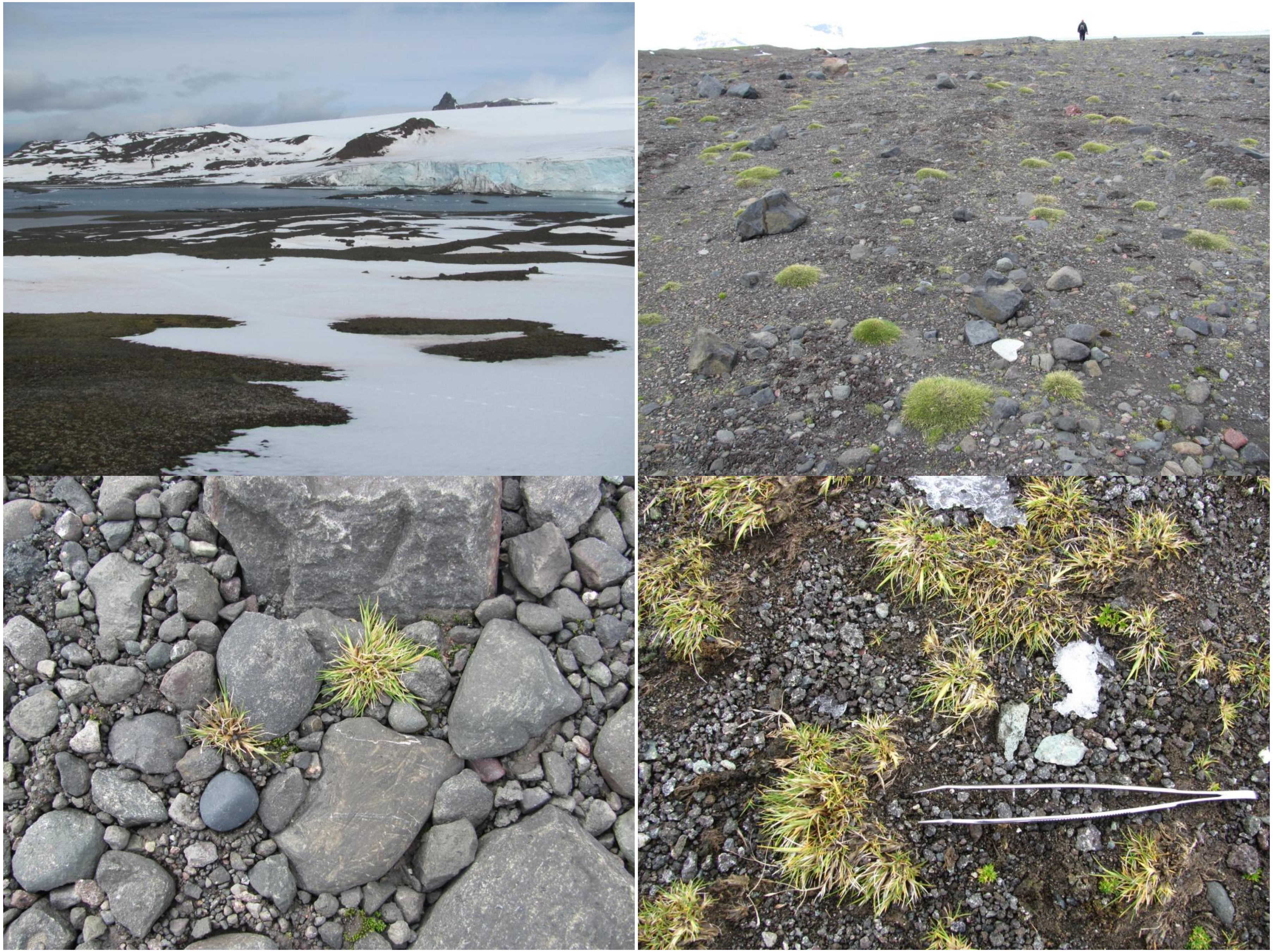

Population #8

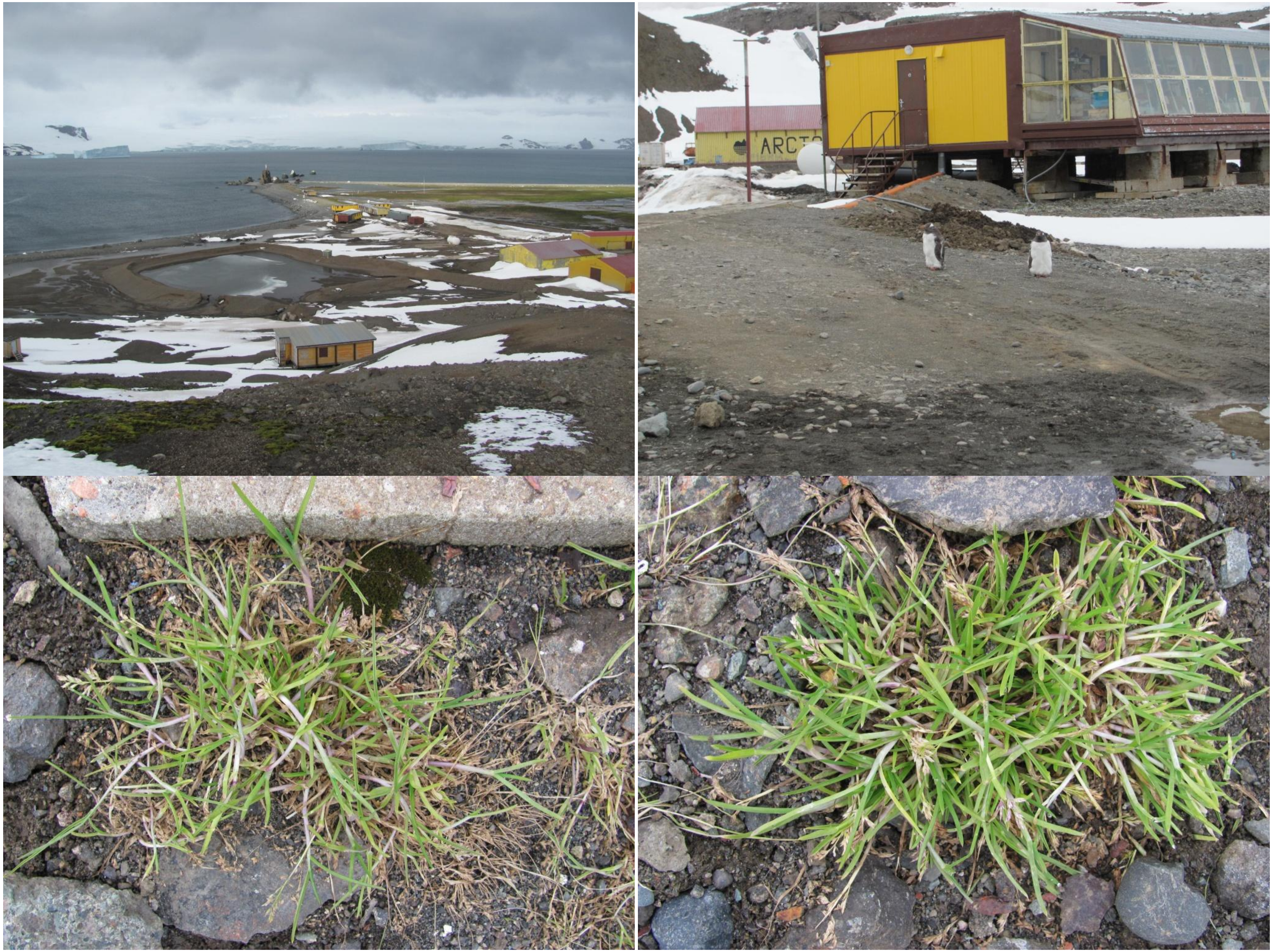

Population #9

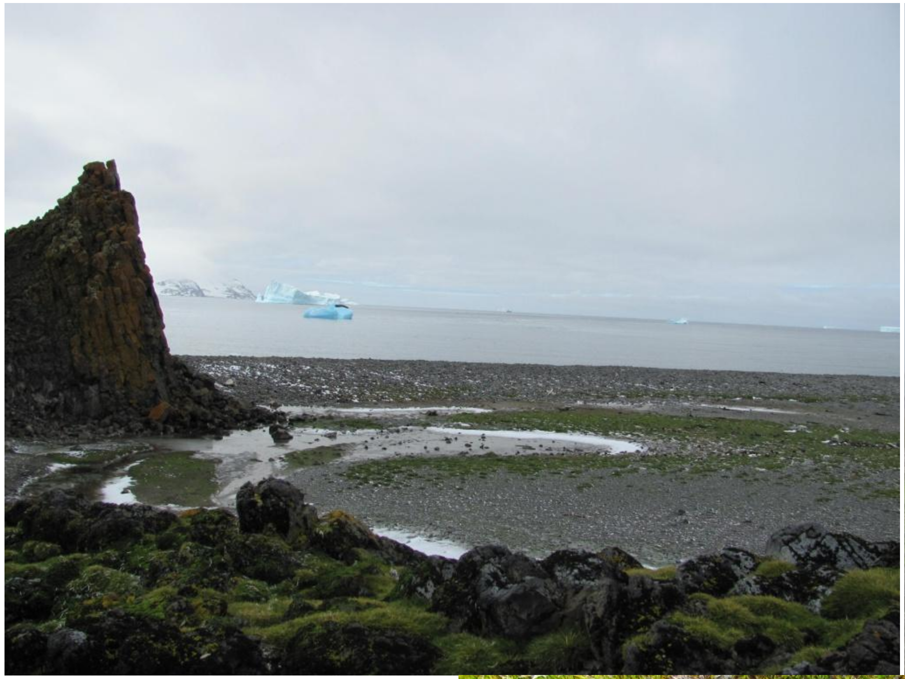
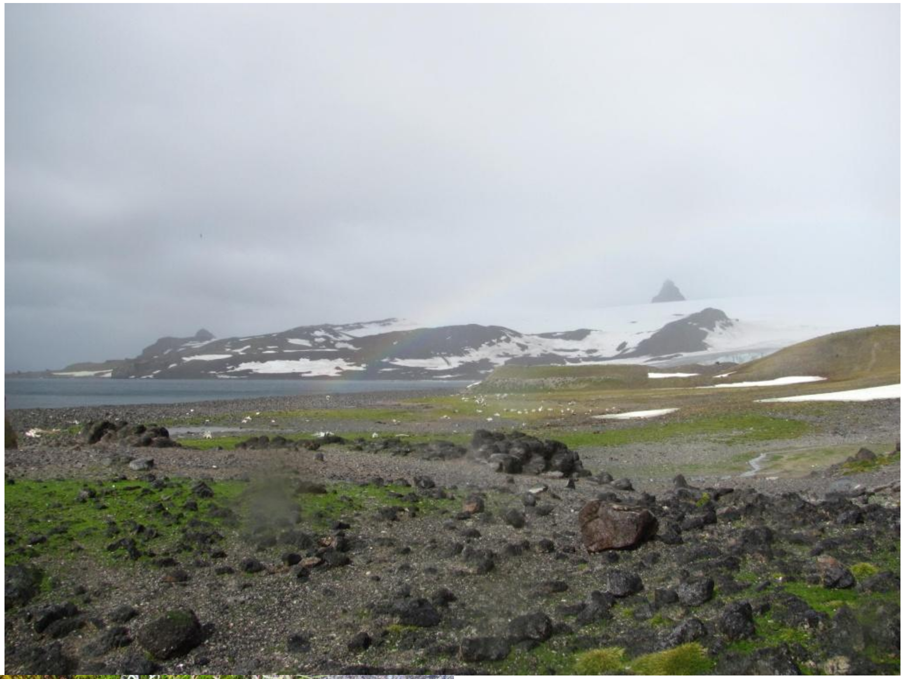
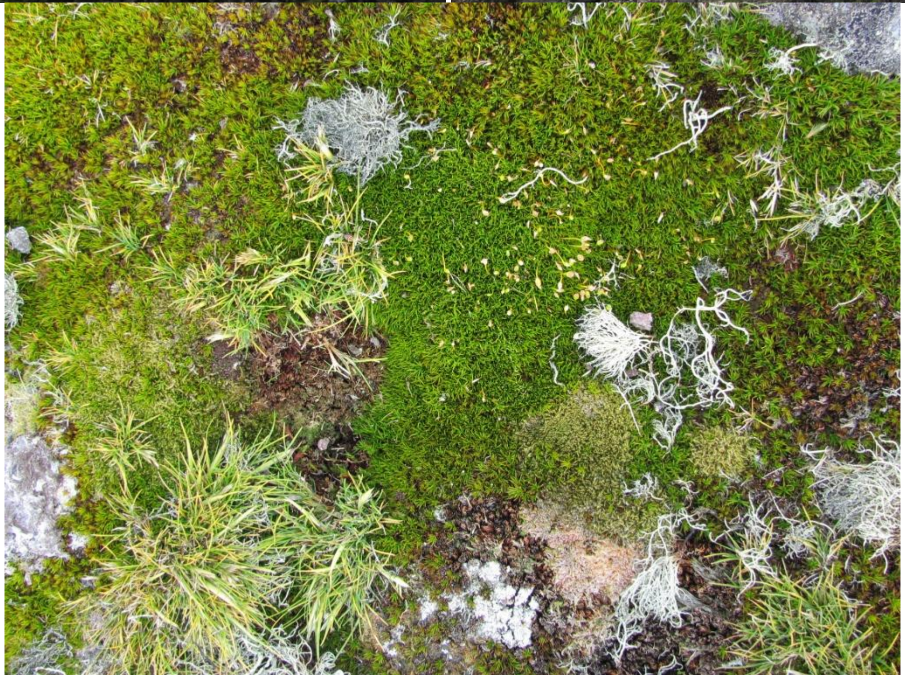

Supplement: Supplementary file 1 — Table S1 [file ECE3-11-648-s001.pdf]
